# Self-Cleaning Alginate–PVA Hydrogel Evaporator with Enhanced Solar Desalination Efficiency and Long-Term Salt Resistance

**DOI:** 10.1021/acsomega.5c05414

**Published:** 2025-10-29

**Authors:** Muhammad F. Siddique, Farag K. Omar, Muhammad Waseem, Ali H. Al-Marzouqi

**Affiliations:** † Mechanical and Aerospace Engineering Department, College of Engineering, 11239 United Arab Emirates University, 15551 Al Ain, United Arab Emirates; ‡ Chemical and Petroleum Engineering Department, College of Engineering, 11239 United Arab Emirates University, 15551 Al Ain, United Arab Emirates

## Abstract

A low-cost, photothermally efficient hydrogel evaporator was fabricated using sodium alginate, poly­(vinyl alcohol), graphite, and multiwalled carbon nanotubes (MWCNTs) via a simple foaming–freezing–ionic cross-linking method. The optimized hydrogel (H2) achieved a high evaporation rate of 3.1 kg/m^2^·h with a solar-to-vapor efficiency of 89% under 1 sun irradiation. XRD analysis showed a reduced crystallite size (9.7 ± 0.3 nm), which was attributed to the improved pore structure and water transport characteristics. SEM images and porosity measurements revealed an interconnected porous network with 85% porosity, supporting capillary flow. The hydrogel exhibited sustained performance at high salinity (>2.1 kg/m^2^·h at 20 wt % NaCl), and after 20 brine cycles, its efficiency loss was <3.3% with no visible salt accumulation. ICP-MS analysis confirmed >99% salt ion rejection using real seawater samples, while compression tests showed a higher compressive stress (0.233 MPa) due to MWCNT reinforcement as compared to that without MWCNTs (0.192 MPa). Statistical *t* tests confirmed significant performance differences between hydrogel variants. The fabrication cost of the system was estimated to be approximately 5.5 USD/m^2^, notably lower than several existing systems (over 40 USD/m^2^). These findings underscore the hydrogel’s potential for decentralized solar desalination owing to its strong photothermal response, mechanical durability, self-cleaning capability, and low production cost. Future work will explore large-scale implementation and further pore structure optimization.

## Graphical abstract

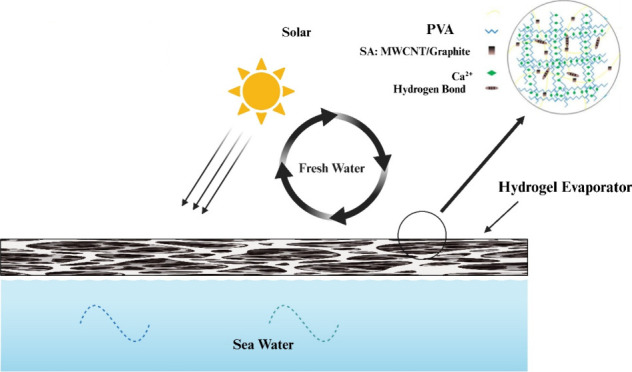

## 1 Introduction

Freshwater scarcity is an escalating global concern, with over 40% of the world’s population having limited access to clean water due to inadequate infrastructure, economic barriers, and climate-related pressures.^1–3^ By 2050, an additional two billion people in vulnerable regions may face water shortages, exacerbated by deforestation, rising sea levels, and loss of wetlands.^2,4^ Although desalination methods such as reverse osmosis and thermal distillation are widely used, they remain costly and energy-intensive,^5,6^ particularly for decentralized or off-grid communities. Solar-driven interfacial evaporation has emerged as a promising alternative, which leverages solar energy to vaporize water at the air–liquid interface with minimal energy input.^7–9^ However, many existing solar evaporator systems face key limitations, including salt accumulation, structural degradation, and complex or unsustainable fabrication processes.^10–12^

Hydrogels, with their three-dimensional porous networks and high-water retention capacity, are ideal candidates for solar evaporators. Recent studies have demonstrated their potential in enhancing the efficiency of evaporation. For instance, Zhou et al.^13^ reported a polypyrrole-based hydrogel with an evaporation rate of 3.6 kg/m^2^·h with a maximum energy efficiency of 92%. Additionally, Sun et al.^14^ developed a β-cyclodextrin-modified polyacrylamide hydrogel that achieved 98% energy efficiency with an evaporation rate of 2.65 kg/m^2^·h for solar-powered water purification. In another paper, Wang et al.^15^ reported a scalable, Porifera-inspired porous hydrogel sponge that achieved an evaporation rate of 2.8 kg/m^2^·h. This sponge could maintain long-term stability (six months) when used for solar desalination, achieving a conversion efficiency of 94%. Li et al.^16^ demonstrated an MXene-integrated hydrogel evaporator with an evaporation rate of 3.4 kg/m^2^·h and efficiency of 85.2% under 1 sun irradiation, highlighting the role of photothermal nanomaterials in realizing performance improvements. Jeong and co-workers^17^ developed a bio renewable polymer-based porous hydrogel with salt/pH tolerance, achieving an evaporation rate of 3.17 kg/m^2^·h and efficiency of 83% under 1 sun irradiation for durable solar desalination. Sun et al.^18^ introduced a portable and eco-friendly hydrogel with strong environmental stability and resistance to contamination, which achieved a solar evaporation rate of 1.61 kg/m^2^·h and conversion efficiency of 55% under standard 1 sun conditions for seawater desalination. In another study, Zhang et al.^19^ reported a straightforward fabrication method of hydrogel-coated surfaces exhibiting strong antifouling and salt-resistant properties and achieving a high-water evaporation rate of 3.64 kg/m^2^·h and conversion efficiency of 90.13% under 1 sun illumination.

Sodium alginate (SA), originating from brown algae, is a naturally occurring anionic copolymer composed of two monomer units, β-d-mannuronic acid (M) and α-L-guluronic acid (G), joined by (1–4) glycosidic bonds. Poly­(vinyl alcohol) (PVA) is a water-soluble, long-chain polymer produced by hydrolyzing polyvinyl esters, typically polyvinyl acetate.^20^ SA and PVA hydrogels can be easily synthesized using calcium ion (Ca^2+^) cross-linking and freeze–thaw techniques, respectively. Ca^2+^ ions interact with guluronic acid blocks in alginate through coordination bonding, forming stable junction zones that reinforce the hydrogel network.^21^ This enhances the mechanical integrity and ionic responsiveness of the system without the need for using chemical cross-linkers.^22,23^

Although considerable progress has been made toward developing hydrogel-based evaporators with improved evaporation rates and energy conversion efficiencies, many reported systems still face significant limitations. Most notably, the fabrication methods are often costly and chemically intensive, involving harmful reagents.^24^ Additionally, issues such as salt accumulation and mechanical degradation under prolonged operation continue to hinder long-term performance and scalability.^25,26^

While several studies have explored photothermal hydrogels for solar steam generation, trade-offs between efficiency, salt resistance, environmental safety, and fabrication scalability still have to be made. Some systems rely on toxic solvents or chemical cross-linkers that complicate processing and reduce environmental compatibility.^14,27^ Others, while achieving high evaporation rates, struggle with salt accumulation or limited long-term durability in actual seawater conditions.^13,15,28^ To address these limitations, this study combines dual-carbon synergy (graphite+MWCNTs) within a physically cross-linked SA/PVA matrix using a simple, nontoxic foaming–freezing–cross-linking method. This approach achieves high evaporation efficiency, intrinsic self-cleaning salt resistance, and strong mechanical durability across 20 desalination cycles, all while using scalable and environmentally benign materials and processes.

## 2 Materials and Methods

### 2.1 Materials

Sodium alginate (SA, molecular weight: 12,000–40,000 Da), poly­(vinyl alcohol) (PVA, alcoholysis degree: 87–89%), calcium chloride, and sodium dodecylbenzenesulfonate (SDBS) were obtained from Sigma-Aldrich. Graphite powder with a purity of 99.99% was obtained from Thermo Scientific. Hydrochloric acid (35–38%), nitric acid (65%), and sulfuric acid (98%) were obtained from SDFCL, Eurolab, and ROMIL - Pure Chemistry, respectively. Multiwalled carbon nanotubes (MWCNTs) with a diameter of 50–90 nm were also obtained from Sigma-Aldrich. Every chemical used was of analytical grade and did not require any additional purification. Distilled water was used to perform dilutions for all the solutions obtained from Bio-Chemix with pH between 6.5 and 7.2.

### 2.2 Characterization

Scanning electron microscopy (JEOL/EO SEM) was used to determine the morphology and structure of the hydrogels. Fourier transform infrared (FTIR) spectroscopy was performed using an FTIR spectrophotometer (FTOR-700, JASCO) with a wavenumber between 500 and 4000 cm^–1^. X-ray diffraction (XRD) was carried out using a Malvern Analytical X’Pert^3^ Powder X-ray diffractometer. Unless otherwise stated, all samples were oven-dried at 60 °C prior to characterization to ensure consistency and reproducibility of results. A contact angle meter (Kyowa Interface Science Ltd., Japan) was used to determine the water absorption time of the hydrogel. An FLIR One Pro thermal camera was used to capture thermal images of the hydrogel during the experiment. The camera’s specifications include a measurement temperature range of −20 to 400 °C, a thermal sensitivity <70 mK, an infrared resolution of 60 × 80 pixels, a measurement accuracy of ±3 °C or ±5% of the reading, and a spectral range of 8–14 μm. A remote temperature gun (Fluke 68 IR thermometer) was used to measure the temperature during the experiment. For irradiation measurements, a solar power meter (SM 206-Solar) was used. For humidity measurements, the Extech RHT20 humidity/temperature datalogger was used. Agilent 7850 ICP-MS was used to measure the ion concentration of purified water and seawater. Finally, the mechanical compression test was performed using an electromechanical universal testing machine.

### 2.3 Solar Vapor Generation Experiment

All experiments for generating simulated solar vapor were carried out in a controlled laboratory setting with an ambient temperature of 23.5 °C and a relative humidity of approximately 38%. To separate the evaporators from the bulk water in the beaker, cylindrical hydrogel samples (4 cm in diameter and 0.8 cm in thickness) were positioned on top of a layer of polyethylene (PE) foam. Water was supplied to the hydrogel evaporator indirectly through cotton balls that connected the hydrogel to a water reservoir. To minimize heat loss, the beaker was wrapped in aluminum foil and insulated with PE foam. The entire apparatus was placed on an analytical balance (Ohaus PR Series) for accurate mass measurements. Illumination was provided by a solar simulator equipped with a xenon lamp, while a solar radiation detector (SM206-Solar) was used to calibrate the radiation intensity. The surface temperature of the hydrogels was recorded using an infrared thermal camera (Flir One Pro).

### 2.4 Preparation of Hydrogels

The hydrogel fabrication protocol was developed by modifying a previously established method.^29^ Key parameters were optimized, including the substitution of sodium dodecyl sulfate with sodium dodecylbenzenesulfonate (SDBS) as a foaming agent, the concentration of photothermal fillers (graphite and MWCNTs), the freezing duration, and the sequence of the foaming–freezing–cross-linking steps. To prepare the hydrogel, SA/PVA/MWCNT+Graphite was dissolved in a beaker with 15 mL of water and stirred at 1600 rpm for 3 h at 95 °C using a magnetic stirrer. SDBS was then added to the solution until bubble formation was observed, continuing for 30 min. The solution was then allowed to cool to room temperature and frozen at −20 °C for 16 h. Following the freezing process, the hydrogel was removed from the refrigerator, and cross-linking was induced by adding a 10% (w/v) calcium chloride (CaCl_2_) solution to the frozen hydrogel. Finally, the hydrogel was soaked in deionized water for 24 h to eliminate any remaining impurities. The freezing step was performed prior to ionic cross-linking to induce phase separation and facilitate the formation of a porous internal network. Ice crystals formed during freezing served as pore-forming templates. After thawing, the structure stabilized through ionic cross-linking with Ca^2+^ ions, preserving the pore architecture.^30^ Table 1 presents the compositions of the different hydrogels prepared. The preparation process is shown in Figure 1.

**Table 1:** Compositions of Hydrogels

name	SA (grams)	PVA (grams)	SDBS (grams)	graphite (grams)	MWCNT (grams)	CaCl_2_ concentration (%w/v)
H0	0.8	0	0.1	0	0	10
H1	0.8	0	0.1	0.15	0.15	10
H2	0.8	0.3	0.1	0.15	0.15	10
H3	0.8	0.6	0.1	0.15	0.15	10

**Figure 1:**
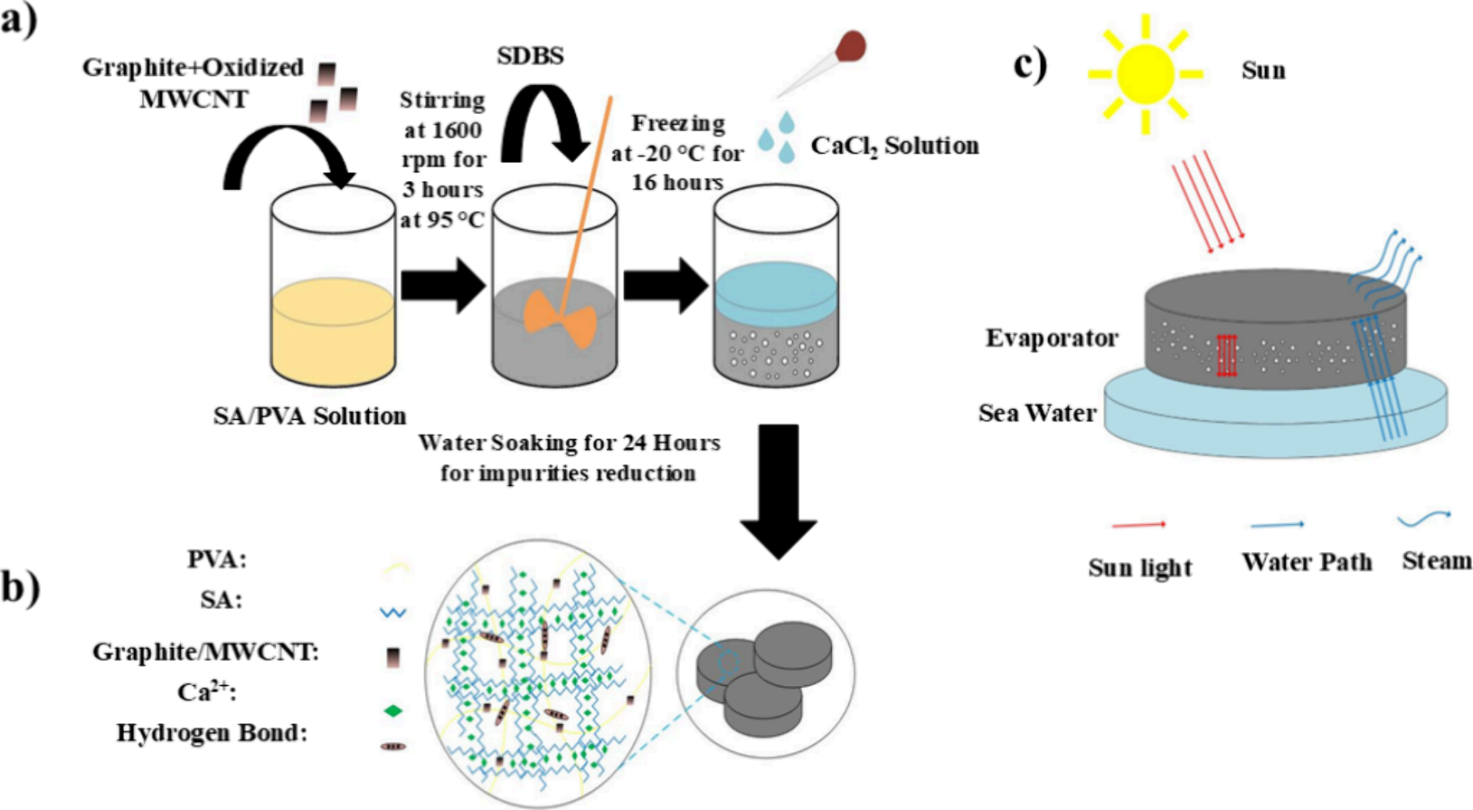
Fabrication and design of SA/PVA hydrogel evaporators. (a) Stepwise manufacturing process: (i) solution mixing of SA/PVA with graphite+MWCNT, (ii) foaming with SDBS surfactant, (iii) freezing at −20 °C for 16 h, and (iv) Ca^2+^ ionic cross-linking. (b) Structural schematic showing interconnected porous network with embedded photothermal fillers (graphite+MWCNT). (c) Operational concept: solar energy absorption (90% broadband efficiency) driving interfacial evaporation, with macro/micropores enabling continuous water supply and salt rejection.

#### 2.4.1 Carbon Nanotube Activation

A 50 mL solution was prepared by mixing 12.5 mL of nitric acid (HNO_3_) and 37.5 mL of sulfuric acid (H_2_SO_4_). The required amount of MWCNTs was oxidatively functionalized using this 3:1 H_2_SO_4_/HNO_3_ mixture to improve dispersibility. The solution was stirred for 3 h at 50 °C and 720 rpm to ensure proper dispersion of the MWCNTs. The MWCNTs were then filtered out and rinsed thoroughly with deionized water to remove any residual impurities. The filtered MWCNTs were then dissolved in a 1 M HCl solution prepared in a 50 mL beaker. The mixture was stirred for an additional 3 h, after which the MWCNTs were filtered once again to complete the purification process. The preparation method is depicted in Figure S1.

## 3 Results and Discussion

### 3.1 Characterization of Hydrogels

The microstructural morphology of hydrogels is crucial to their performance, as porosity influences their water adsorption capabilities.^31^ Figure 2 presents SEM images of hydrogels H0, H1, H2, and H3, highlighting significant variations in their pore structure and distribution, influenced by differences in composition and processing. In the case of H0 and H1, the pores are narrow and unevenly distributed, primarily due to the absence of PVA in their formulation. PVA typically acts as a structural stabilizer by forming a cross-linked skeleton during the freezing process, which helps maintain the integrity and distribution of pores. The lack of PVA in H0 and H1 resulted in insufficient structural support, causing the pores to collapse during the thawing stage. Additionally, the cross-linking reaction between SA and CaCl_2_ contributed to a reduction in the pore volume, further exacerbating pore collapse. These combined factors resulted in the observed narrow, irregular pore structures, a phenomenon also documented in previous studies.^29^ More detailed SEM images at various magnifications are provided in Figures S2–S5 to offer a more comprehensive view of the surface morphology, pore distribution, and internal structure of the hydrogel samples (H0, H1, H2, and H3). These images further confirm the progressive development of a highly porous and interconnected network.

**Figure 2:**
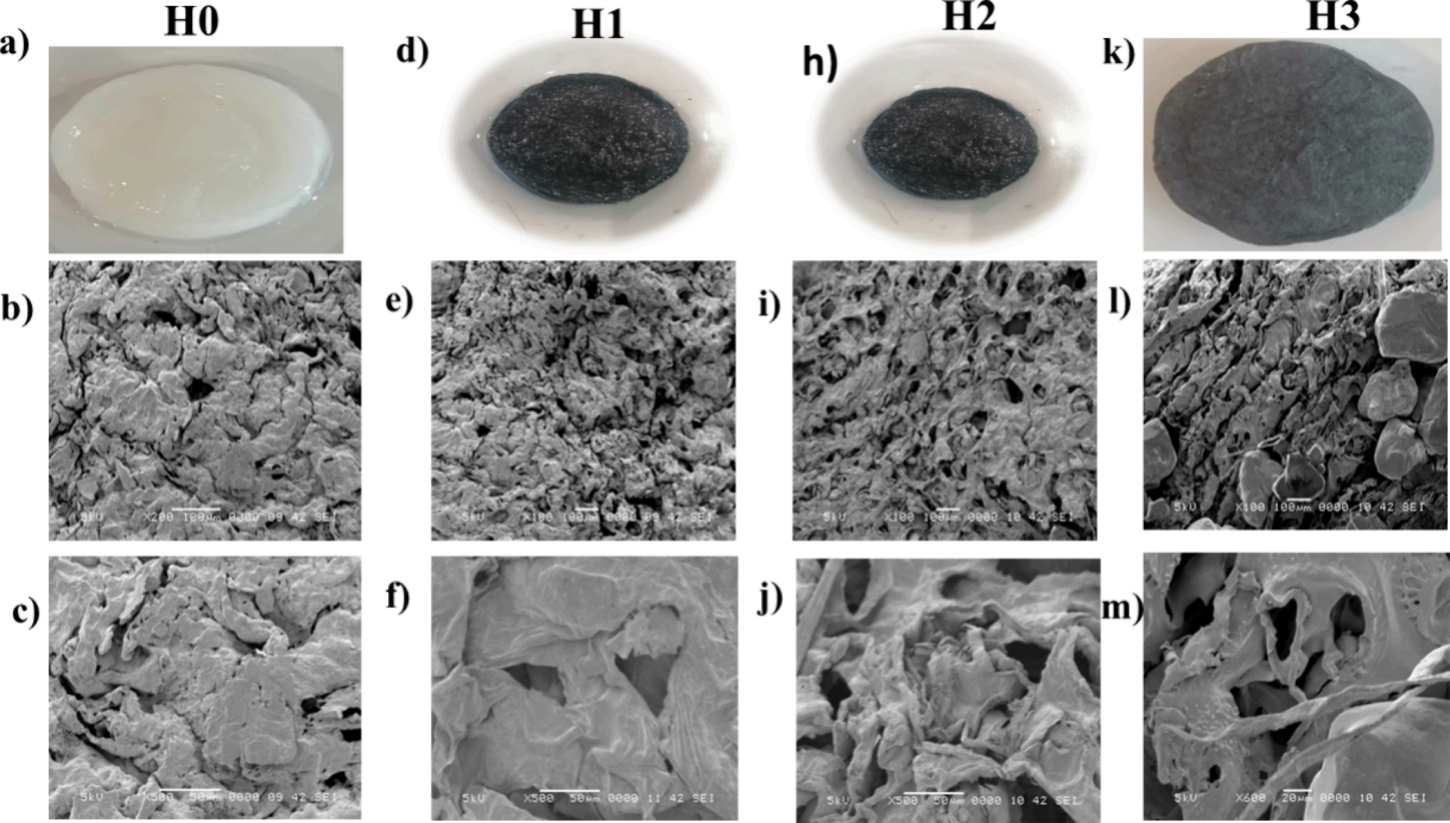
SEM images of hydrogels (a–c) H0, (d–f) H1, (h–j) H2, and (k–m) H3, illustrating pore morphology variations. H2 (h–j) exhibiting uniform, open pores (>100 μm) due to PVA stabilization, and H0/H1 exhibiting collapsed pores from insufficient cross-linking. Excessive PVA in H3 (k–m) leading to an uneven pore distribution.

Quantitative pore analysis was performed on the SEM micrographs using ImageJ software to evaluate the pore size and distribution across different hydrogel samples. The H0 hydrogel exhibited small and irregular pores with an average diameter of 22 μm, suggesting limited water transport pathways. Meanwhile, H1 showed slightly larger and more defined pores with an average diameter of 36 μm. Notably, the optimized H2 hydrogel demonstrated a well-interconnected porous network, with pore sizes ranging from 35 to >100 μm and an average diameter of 78 μm, indicating a moderately uniform distribution beneficial for capillary water transport and vapor diffusion. In contrast, H3 had a more compact structure with reduced pore openness and a lower average pore size of 59 μm due to hindered pore formation during freezing. In comparison, H2 displayed a significantly improved porous structure, characterized by larger and more uniform pores. A closer examination revealed that most of these pores were open, providing an interconnected network highly advantageous for water adsorption and transport. The incorporation of PVA in H2 was instrumental in achieving this enhanced porous structure. PVA’s role in stabilizing the pore network during the freezing–thawing process prevented collapse and ensured a homogeneous distribution of pores, resulting in a robust and effective porous matrix. However, in H3, the pore structure deviated from the optimal characteristics observed in H2. This inconsistency in pore structure was attributed to the excessive addition of PVA, which disrupted the formation of a uniform porous network during the freezing step. The imbalance in polymer composition interfered with phase separation, resulting in a denser internal structure with limited pore connectivity. These findings highlight the sensitive balance required in the formulation process, as both insufficient and excessive PVA content can negatively impact the final pore structure. An optimal balance is essential to achieve a porous structure that maximizes water adsorption while maintaining structural integrity. This foaming–freezing–ionic cross-linking approach directly addresses the toxic cross-linker limitation.^24^ Unlike glutaraldehyde-cross-linked systems, our Ca^2+^ method achieves improved porosity without using hazardous reagents.^25^

To quantify the porosity of the hydrogels, a liquid displacement method using distilled water was employed.^32^ The geometric volume of each dried sample was calculated from the sample’s physical dimensions, and the solid polymer volume was estimated by measuring the displacement of distilled water. Porosity was then determined using eq S1.

Although ethanol is typically preferred to avoid swelling, distilled water was used under controlled conditions to reflect the hydrogel’s application environment. Figure 3a indicates that a clear increase in porosity could be observed with the incorporation of a certain amount of PVA.

**Figure 3:**
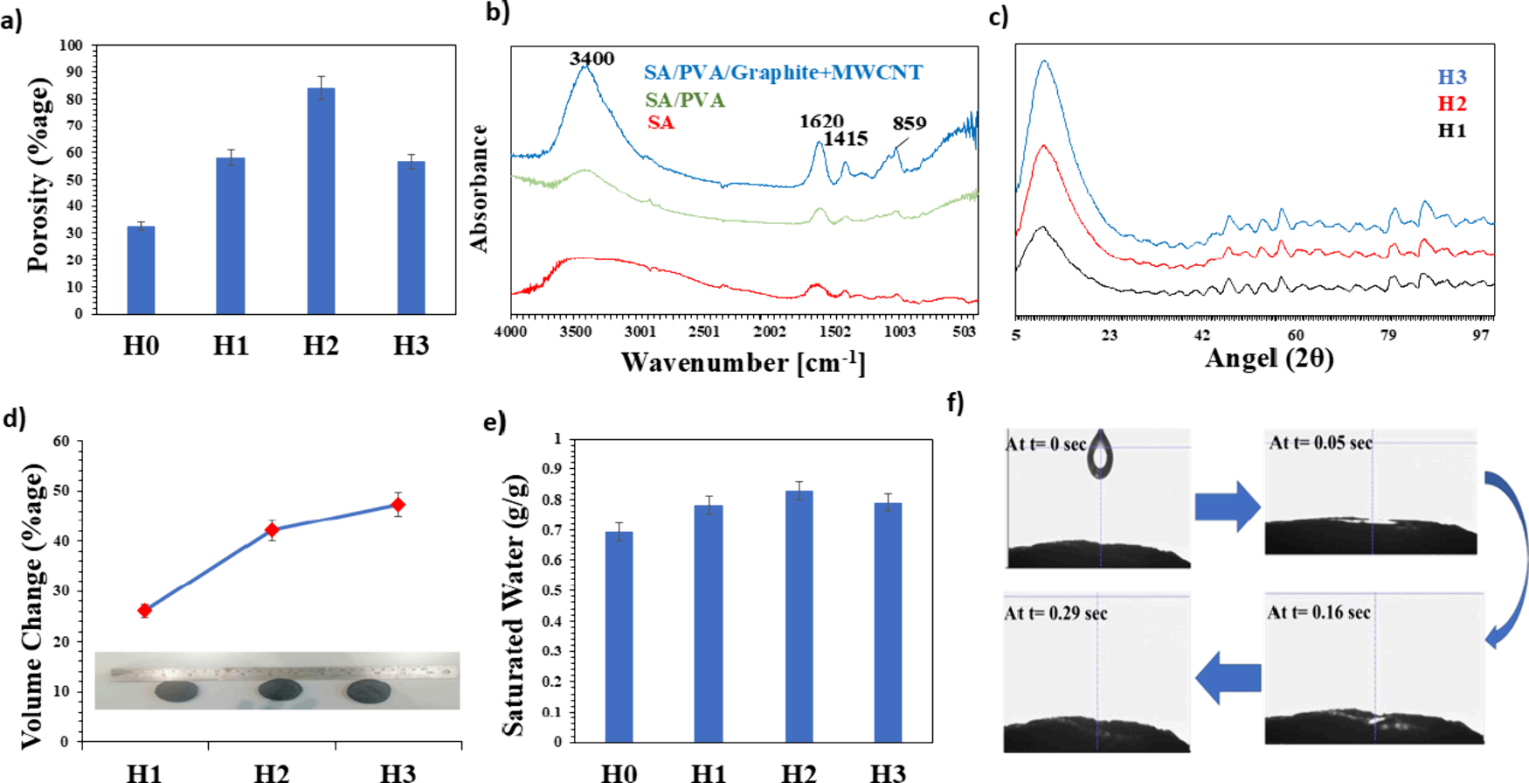
(a) Porosity of hydrogels measured by liquid displacement using distilled water. (b) FTIR spectra of SA, SA/PVA, and SA/PVA/graphite-MWCNT composites, showing shifts in O–H (3400 cm^–1^) and C=O (1620 cm^–1^) bonds due to cross-linking. (c) XRD patterns of H1–H3 hydrogels, with H3 exhibiting the highest crystallinity (peak at 19.8°). (d) Volume expansion of dry hydrogels after immersion in water for 12 h, with H2 showing optimal swelling. (e) Saturated water content, with H2 achieving 0.83 g/g absorption. (f) Water adsorption test, with H2 absorbing 1 μL water in 0.29 s.

Figure 3b presents the FTIR spectra of three composites in absorbance mode. The FTIR spectra of the three materials (SA, SA/PVA, and SA/PVA/Graphite+MWCNT) offer valuable insights into the chemical composition and interactions of the composite materials. In the spectrum of SA, the broad peak around 3400 cm^–1^ corresponds to intra- and intermolecular bonding due to O–H stretching vibration, which is characteristic of the hydroxyl groups present in SA.^33^ Additionally, the strong band at approximately 1620 cm^–1^ is attributed to the asymmetric stretching of the carboxylate (−COO^–^) group, while the weaker band around 1415 cm^–1^ corresponds to symmetric COO^–^ stretching. These peaks are typically associated with alginate’s carboxyl groups and are key indicators of Ca^2+^ ion coordination bonding. Upon cross-linking with CaCl_2_, shifts in the 1620 and 1415 cm^–1^ bands are observed in the SA/PVA and SA/PVA/Graphite+MWCNT spectra, indicating the formation of coordination complexes between Ca^2+^ ions and the guluronic acid blocks of alginate.^34^ In the SA/PVA spectrum, the broad O–H stretch near 3200–3500 cm^**–**1^ becomes more pronounced, which is likely due to the hydrogen bonding interactions between the hydroxyl groups of both SA and PVA. This suggests that the two polymers form a composite structure through intermolecular interactions. Additionally, the 1600 cm^–1^ peak also shows a slight shift, suggesting the formation of hydrogen bonds or other types of interactions between the carboxyl groups of alginates and the hydroxyl groups of PVA, thus contributing to the overall stability and cohesion of the composite. The 1400–1500 cm^–1^ region indicates the presence of PVA through C–H bending vibrations, further confirming its successful incorporation into the hydrogel matrix. The SA/PVA/Graphite spectrum reveals distinct changes, particularly in the 3200–3500 cm^–1^ region, where the broad O–H stretching peak becomes less pronounced.^35^ This could be attributed to the interaction of graphite with the hydroxyl groups, leading to reduced availability of free hydroxyl groups in the composite. Additionally, new peaks or intensity shifts in the 500–900 cm^–1^ range can be observed, which are likely due to the vibrational modes of the graphite, including C=C stretching or skeletal vibrations typical of graphite and graphene layers.^36^ These changes indicate that graphite is well incorporated into the composite structure, potentially enhancing its thermal and electrical properties. The overall FTIR analysis demonstrates that the incorporation of PVA and graphite into the SA matrix significantly changes the chemical structure, suggesting strong interactions among the components. This synergy between the materials enhances the hydrogel’s structural and functional properties.

Figure 3c shows the XRD results of hydrogels H1, H2, and H3. The XRD graph presents the diffraction patterns of the hydrogel samples, highlighting distinct variations in their crystalline structures. Sample H3 exhibited the highest peak intensities, with a prominent diffraction peak around 2θ ≈ 19.8°, indicating the highest degree of crystallinity. This peak corresponds to the (101) plane of semicrystalline PVA, commonly reported in SA/PVA-based hydrogels.^36,37^ In contrast, H2 showed a lower peak intensity at the same position, suggesting a more amorphous structure due to reduced PVA content or disruption of ordered domains by carbon nanofillers.^38^ The differences in the peak intensities and sharpness reflect the impact of synthesis conditions and differences in compositions, such as differences in PVA content, which is known to significantly influence both crystallinity and swelling behavior.

Although X-ray photoelectron spectroscopy (XPS) and Raman analyses could not be performed due to a lack of instrumentation, such studies would provide important complementary evidence. XPS could confirm Ca^2+^–alginate coordination through shifts in O 1s/C 1s binding energies and identify oxygenated groups on functionalized MWCNTs, validating polymer–filler interactions.^39^ Raman spectroscopy could reveal the characteristic G band (∼1580 cm^–1^, sp^2^ C=C stretching) and D band (∼1350 cm^–1^, defect-induced), with the I_D_/I_G_ ratio indicating defect density and dispersion quality of the carbon fillers.^40^ These insights could strengthen the correlations already observed between microstructure, water transport, and photothermal efficiency.

The crystallite size of the hydrogels was analyzed to quantify the structural features influencing solar desalination performance. The average crystallite size (*D*) was calculated using the Debye–Scherrer equation (eq S2).^41^ The analysis revealed that H3, which contained the highest PVA content, exhibited the largest crystallite size (18.2 ± 0.5 nm) and enhanced crystallinity due to increased polymer chain ordering. In contrast, H2 showed smaller crystallites (9.7 ± 0.3 nm), suggesting a balance between crystalline and amorphous regions that facilitated its interconnected porous structure and optimal water transport properties. Because of a lack of PVA, H1 displayed an intermediate crystallite size (12.1 ± 0.4 nm), consistent with the crystalline domains of the alginate matrix.^42^ These findings correlate well with the SEM observations (Figure 2) and evaporation performance (Figure 5), where H2’s intermediate crystallinity and pore structure contribute to its enhanced desalination efficiency.

The pore structure of hydrogels is mostly determined by their volume shrinkage, with a large shrinkage resulting in decreased porosity. When cross-linking pure SA hydrogels with Ca^2+^ ions, ionic cross-links are formed, increasing intermolecular interactions and causing SA chains to consolidate into a hydrogel. This causes noticeable volume shrinkage. However, the addition of PVA chains creates a network of supporting polymers that negates this effect. During the hydrogel production process, the PVA framework maintains the pore structure and reduces the amount of shrinkage caused by Ca^2+^ SA cross-linking. The enhanced evaporation performance is attributed to the more visibly interconnected porous structure observed in the SEM images, as well as the high porosity confirmed through the liquid displacement measurements, which together promote more efficient water transport pathways. The percentage volume increase of hydrogels is shown in Figure 3d, after immersing the completely dry hydrogel in pure water for 12 h. According to the graph, the PVA content increases in the hydrogel volume percentage.

By analyzing the saturated water content of the hydrogels, their internal pore structure and water transport properties can be assessed. The saturated water content (*Q*_s_) was calculated using eq S3. As shown in Figure 3e, H0 exhibited the lowest water absorption capacity among the hydrogels, with a value slightly below 0.7 g/g. H1 demonstrated a moderate increase, absorbing approximately 0.79 g/g. H2 showed the highest water absorption capability, with a value of approximately 0.83 g/g. Meanwhile, H3 absorbed slightly less water than H2, with a value close to 0.8 g/g. A progressive increase in water absorption could be observed from H0 to H2, followed by a slight decline with H3. The large amount of water absorption in H2 and H3 is due to pores that saturate them more than H0 and H1. As H0 could not sustain the significant water due to a lack of porosity, it was not considered for further investigation. Meanwhile, as H2 displayed a significant water absorption and volume increase, it was used as a specimen for the water adsorption test, as shown in Figure 3f. According to the results, it took 0.29 s for 1 μL to be completely absorbed in H2, proving the high-water absorption performance of H2. To visually assess the water absorption capability of the H2 hydrogel, a video was also recorded (Supporting Information V1). The video was recorded at 16× slower speed to clearly capture the absorption dynamics.

These results confirm that H0 and H1 cannot retain water. Due to a lack of pores, their volume increases when they are fully saturated. However, H2 can retain a high amount of water with a significant volume increase. Therefore, due to their significantly improved sun absorption capacity and effective water transport properties, H2 hydrogels show considerable promise as high-performance solar interface evaporators.

### 3.2 Water Transport Performance of Hydrogels

As water transport performance directly affects the functionality of hydrogels, it is a crucial factor in both their design and use. Maintaining the hydrogel matrix’s structural integrity and functionality under dynamic settings requires quick hydration and dispersion, which is made possible by efficient water transport. Figure 4 demonstrates the water transport experiment. A 0.8 mm-thick piece of cellulose paper was placed on top of a dehydrated hydrogel, with the base of the hydrogel in contact with bulk water. We observed and recorded the rate of water transport from the base to the surface of the hydrogel to visually illustrate its water transport properties. The results made it evident that the H2 hydrogel had an exceptionally high-water transport rate, completely wetting the cellulose paper in only 60 s.

**Figure 4:**
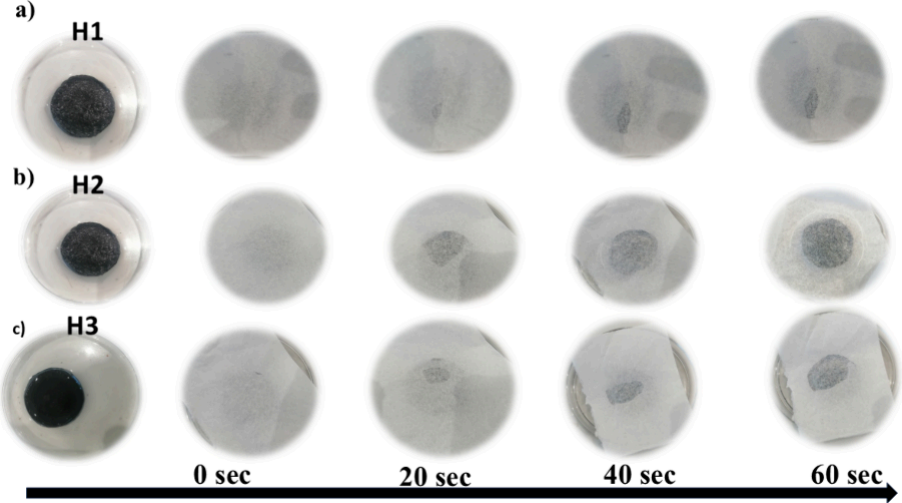
Water transport performance of hydrogels (a) H1, (b) H2, and (c) H3 evaluated by wetting of cellulose paper (thickness of 0.8 mm) placed atop the dehydrated hydrogels. Time-lapse images indicating H2 fully saturating the paper in 60 s and H1 and H3 exhibiting slower transport rates due to their lower porosity.

In comparison, the H0 and H1 hydrogels demonstrated slower water transport rates due to their lower porosity, which limited their ability to effectively channel water to the interface. These findings highlight the excellent water transport capabilities of H2, making it a promising material for applications requiring rapid hydration and efficient water delivery.

### 3.3 Hydrogels’ Ability to Generate Solar Vapor

The evaporation capability of the hydrogels was assessed by solar evaporation experiments. To minimize heat loss from the hydrogels during solar evaporation, PE foam was utilized as an insulating layer between the evaporator and the bulk water. This insulation effectively reduced heat dissipation in the water reservoir. Additionally, hydrophilic cotton was used to facilitate efficient water transport from the bulk water to the evaporator due to its superhydrophilic nature, ensuring a continuous water supply. An extra layer of PE foam and aluminum foil was applied to the outside of the beaker to increase thermal efficiency and shield the entire system from external environmental impacts. The experimental setup, as shown in Figure 5a, was established to evaluate the evaporation capabilities of pure water and the prepared hydrogels. In addition to drastically lowering heat loss, this configuration kept the water flowing continuously to the evaporator. The evaporator was subjected to 1 kW/m^2^ of artificial sunshine for 1 h after the beaker was placed on an analytical balance. The mass change of the beaker (Figure 5b) and the temperature variation on the evaporator surface (Figure 5d,e) were tracked and documented during the evaporation process.

**Figure 5:**
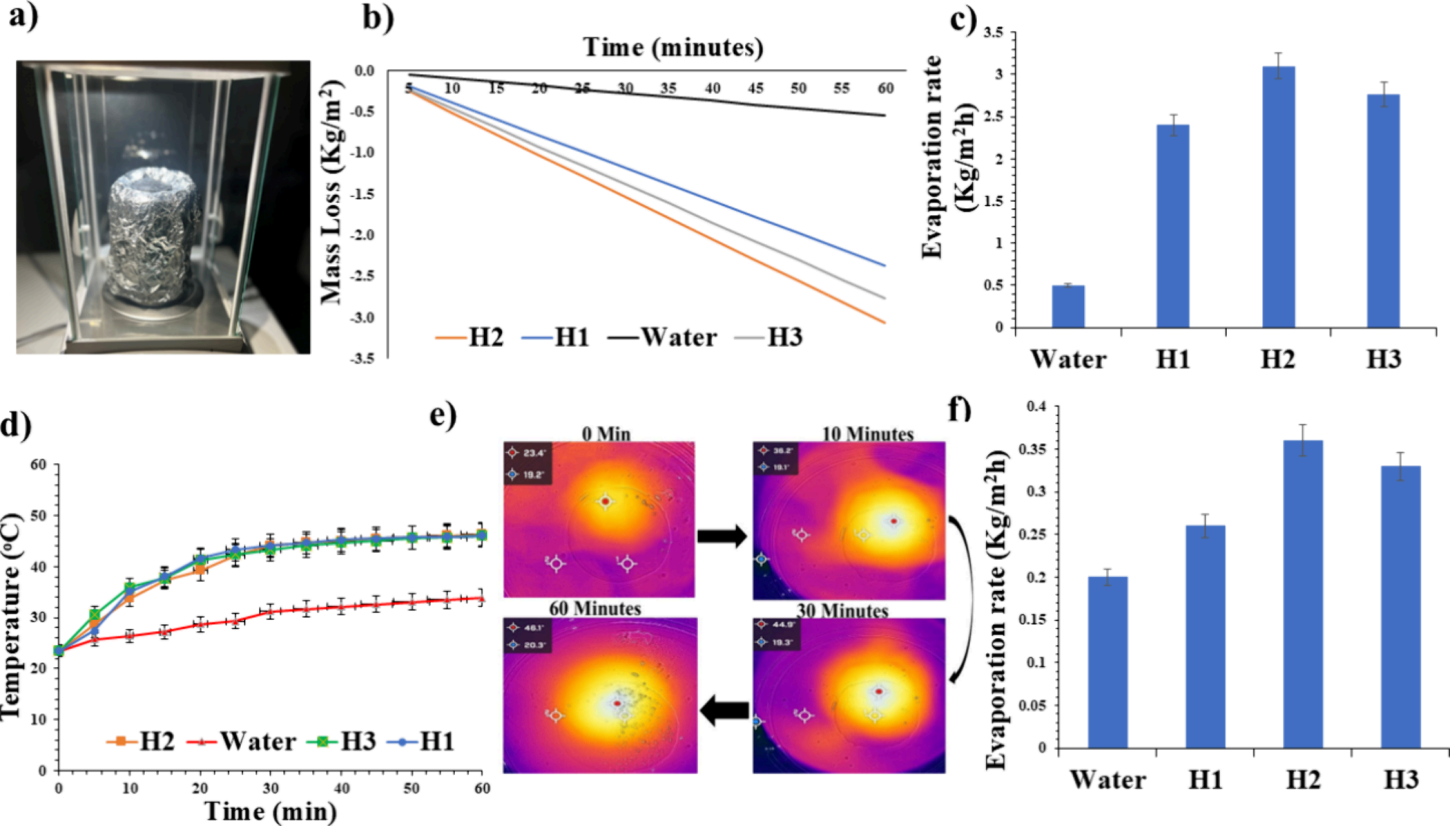
(a) Experimental setup for solar evaporation: hydrogel on insulated foam, with cotton water supply, and solar simulator. (b) Mass loss under 1 sun irradiation, with H2 achieving a mass loss of 3.1 kg/m^2^ (500% > water). (c) Evaporation rates confirming H2’s superiority (3.1 vs 2.4 kg/m^2^·h for H1). (d) Surface temperatures stabilizing at 46 °C for all hydrogels. (e) IR images indicating that H2 shows uniform heating (scale: 19–46 °C). (f) Dark evaporation controls validating solar-driven performance.

Figure 5b presents the mass loss of the hydrogels under one sun irradiation for 60 min, comparing water with hydrogels H1, H3, and H2. Water exhibited the lowest mass loss at 0.5 kg/m^2^, serving as a baseline for natural evaporation under identical conditions. Meanwhile, H1 showed a mass loss of 2.4 kg/m^2^, performing 4.4 times better than water with a 340% increase in evaporation efficiency. H3 achieved a slightly higher mass loss of 2.8 kg/m^2^, representing a 5-fold improvement over water and a 400% increase in efficiency, outperforming H1 by 13.64%. The most efficient material, H2, recorded the highest mass loss at 3.1 kg/m^2^, demonstrating a 6-fold improvement over water and a 500% increase in evaporation efficiency, outperforming H3 by 20% and H1 by 36.36%. The differences in performance stem from variations in the hydrogels’ structural and thermal properties, such as porosity, thermal conductivity, and water retention capacity. These findings highlight the improved performance of H2 in solar-driven water evaporation, making it a promising candidate for applications in solar desalination and water purification technologies.

Figure 5c compares the evaporation rates of water, H1, H2, and H3 under identical conditions, revealing significant differences in performance. Pure water exhibited the lowest evaporation rate at 0.5 kg/m^2^·h, highlighting its limited thermal efficiency under simulated solar irradiation. In contrast, the hydrogels (H1, H2, and H3) demonstrated enhanced evaporation rates due to their efficient water retention, solar absorption, and thermal management properties. H2 achieved the highest evaporation rate at 3.1 kg/m^2^·h, followed by H3 at 2.8 kg/m^2^·h and H1 at 2.4 kg/m^2^·h, due to variations in their structural and thermal characteristics. H2’s enhanced performance suggested optimal porosity and heat localization, whereas H3′s slightly lower performance could be attributed to reduced water diffusion due to its closed pores. Although H1 outperformed water, it exhibited the lowest evaporation rate among the hydrogels, possibly due to its lower solar absorbance and less efficient water transport properties.

To statistically validate the enhanced evaporation performance of the H2 hydrogel, unpaired two-sample *t* tests were conducted comparing H2 with H1 and H3. The results revealed highly significant differences in evaporation rates: for H2 versus H1, *t* = 27.59, degree of freedom (*d*_f_) = 3.43, *p* < 0.0001, and for H2 versus H3, *t* = 11.24, *d*_f_ = 3.53, *p* = 0.0007. These findings confirm that the enhanced water transport and photothermal synergy in H2 are not only observable but also statistically significant. Thus, H2’s performance advantage is robust and unlikely due to random variability. The results are shown in Table S1.

Figure 5d compares the changes in the surface temperature of water and the three hydrogels (H1, H2, and H3) under one sun irradiation for 60 min, starting at an ambient temperature of 23.5 °C. Water exhibited the slowest temperature rise, reaching only 31.2 °C after 30 min and stabilizing at 33.9 °C by the end of the experiment. This indicates limited thermal responsiveness and heat retention under solar irradiation. In contrast, all three hydrogels significantly outperformed water in terms of thermal response. The surface temperature of H1 reached 39.2 °C after 20 min, 42.4 °C after 30 min, and gradually increased to 46 °C by the end of the hour, achieving a 35.7% higher value than that for water after 30 min. H2 showed a similar trend, with its temperature reaching 41.6 °C after 20 min, 44.2 °C after 30 min, and stabilizing at 46 °C by the end of the experiment, representing a 30.7% higher value than that for water at 30 min. H3 demonstrated the most rapid initial rise in temperature, which reached 44 °C after 20 min and 44.1 °C after 30 min, before stabilizing early at 46 °C, making it 41.4% higher than the value for water at 30 min. While all hydrogels reached the same final surface temperature of 46 °C, their heating rates differed. H3 exhibited the fastest initial temperature rise but stabilized early, whereas H1 and H2 showed a more gradual and sustained increase. Compared to water, the hydrogels displayed superior thermal performance as a result of their enhanced solar energy absorption and conversion, which can be attributed to their material properties, such as their higher thermal conductivity and optimized water evaporation mechanisms. Figure 5e presents infrared images illustrating the temperature distribution of H2 at 0, 10, 30, and 60 min under simulated solar irradiation.

Subsequently, eqs S4 and S5 were used to calculate the efficiency of solar evaporation (η).^13,43,44^ To calculate the hydrogels’ equivalent enthalpy of evaporation, dark evaporation tests were carried out to ascertain the dark evaporation rates of both pure water and the hydrogels, as shown in Figure 5f. Eq S6 was used to calculate the enthalpy of water vaporization in the hydrogels. Among the tested materials, hydrogel H2 exhibited the lowest equivalent enthalpy of water vaporization at 1202 J/g, indicating its higher energy efficiency in facilitating water evaporation. In comparison, the equivalent enthalpy values for H1 and H3 were 1373 and 1261 J/g, respectively. According to these results, H2’s enthalpy was 12.4% lower than that of H1’s and 4.7% lower than that of H3′s, highlighting its efficient energy utilization. Additionally, the vaporization efficiency of H2 was calculated to be 89%, surpassing that of H1 (∼78%) and H3 (∼80%), further underscoring its potential as a high-performance material for solar water evaporation applications. The performance of H2 is compared to that of other state-of-the-art hydrogel evaporators in Table 2.

**Table 2:** Comparison of Photothermal H2 Evaporator with Those Designed in Recent Studies

reference	system/study description	evaporation rate (kg/m^2^·h)	conversion efficiency (%)	salt rejection (%)	key features
current study	SA/PVA/graphite + MWCNT	3.10	89	>99	dual-carbon synergy, salt self-cleaning, low-cost fabrication
^13^	polyvinyl alcohol and chitosan and polypyrrole as the light absorber	3.60	∼92		good light absorption, no salt resistance
^14^	β-cyclodextrin-modified hydrogel	2.65	98		high efficiency, multistep synthesis
^15^	porous hydrogel sponge (sponge@PVP/Fe^3+^@TA-1h)	2.8	94	99.99	high porosity, mechanical stability, limited pH stability
^16^	polyvinyl alcohol/biochar hydrogel	1.89	85.2	∼99.9	hierarchical porous structure, limited scalability
^18^	CNF/PVA/MWCNTs hydrogel	1.61	not explicitly stated	∼99.9	biodegradable and porous, high compressibility, limiting scalability
^19^	PVA/CB/TA/Fe^3+^-coated foam	3.64	90.13	effective	antifouling and salt-resistant due to PVA hydration and porous foam
^45^	CMC/chitosan/C-dot hydrogel	1.4	89	>99	biocompatible, low-cost materials, and recyclable
^46^	sodium alginate-polydopamine hydrogel	1.40	not explicitly stated	>99	antibacterial, low-cost, biodegradable materials
^47^	tree root/graphene oxide (DRGO) evaporator	1.60	96.5	>99	natural multiscale channels, super hydrophilic, long-term stability

### 3.4 Salt Resistance of Hydrogel Evaporator

Salt crystal accumulation is a major operational issue for solar evaporators. These crystals can obstruct water transport pathways, diminishing the efficiency of water transfer. Additionally, salt accumulation on the surface of the evaporator can reflect sunlight, thereby lowering the absorption of solar energy. These problems eventually cause the total evaporation rate to drop, which impairs the efficiency of the desalination process. Figure 6a,b show the mass loss and evaporation rate calculated using the H2 Evaporator in 5 wt % brine under 1 kW/m^2^ of simulated solar radiation over a 10 h of evaporation. During the evaporation process, the cumulative mass loss steadily increased, reaching −29.6 kg/m^2^, while the evaporation rate exhibited a gradual decline over time. Starting at 2.9 kg/m^2^·h in the first hour, the evaporation rate peaked slightly at 3.0 kg/m^2^·h by the third hour, before steadily decreasing to 2.8 kg/m^2^·h by the tenth hour. This decline in performance is strongly correlated with the accumulation of salt crystals on the evaporator surface, as evidenced by a visual comparison of the evaporator at the beginning and the end of the experiment. Initially, the evaporator surface was smooth and free of any deposits, allowing efficient water transport and solar energy absorption. Nevertheless, this H2 evaporator performed significantly well, with the evaporation rate decreasing by only 0.1 kg/m^2^.h after 10 h of operation, highlighting its salt resistance properties.

**Figure 6:**
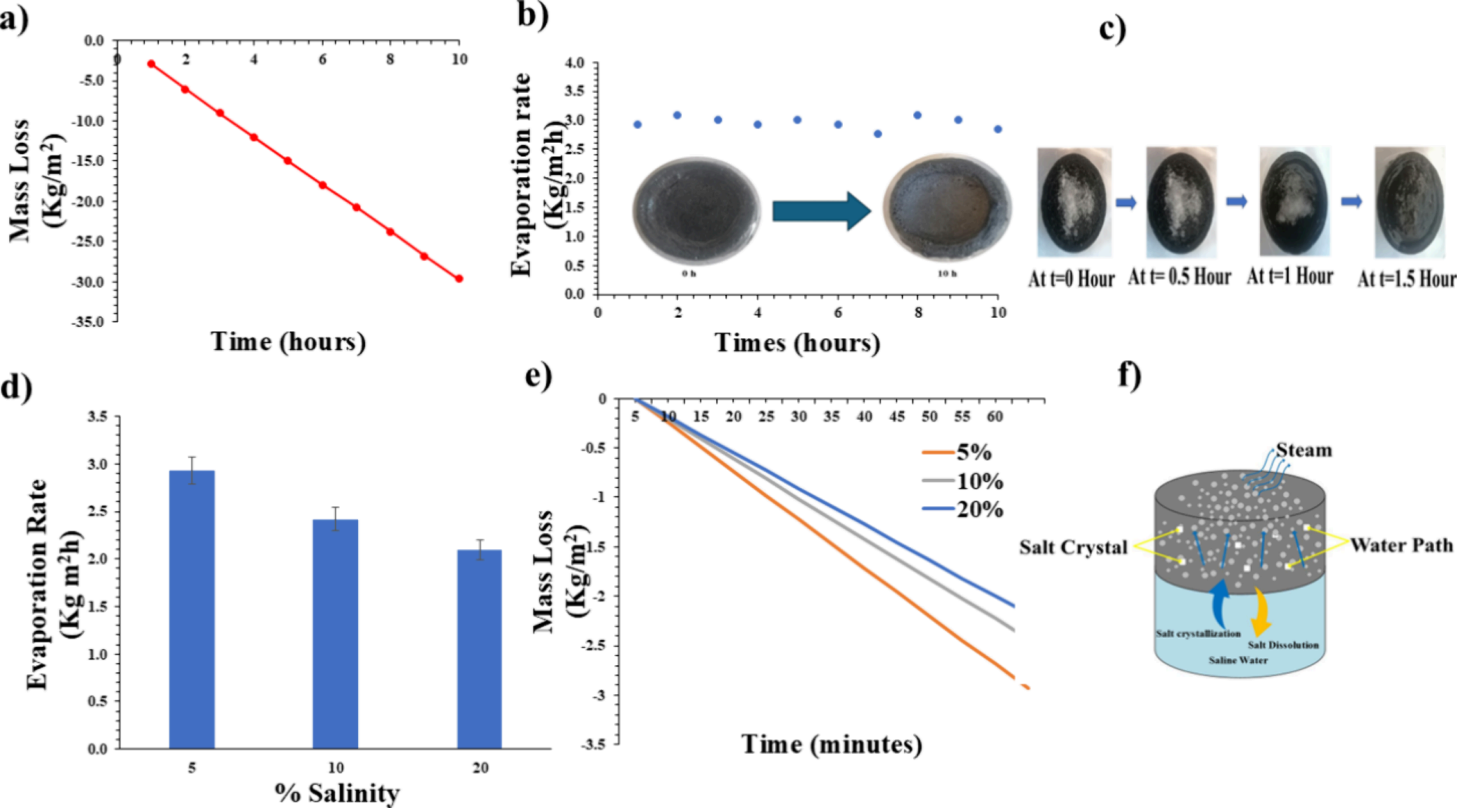
Long-term salt resistance performance of H2 hydrogel evaporator. (a) Cumulative mass loss and (b) corresponding evaporation rates in 5 wt % brine under 1 sun irradiation (1 kW/m^2^) over 10 h, demonstrating stable performance (2.9→2.8 kg/m^2^·h). (c) Time-lapse optical images showing complete dissolution of surface NaCl crystals within 90 min. (d) Evaporation rate and (e) mass loss at varying salinities (5, 10, and 20 wt %), with H2 maintaining 2.1 kg/m^2^·h even at 20 wt % salinity. (f) Proposed salt resistance mechanism: interconnected macro/micropores enabling downward salt ion diffusion while sustaining upward water transport.

To evaluate the desalination performance of H2, solid NaCl particles were intentionally deposited on its surface, and the time required for complete dissolution of the crystalline salt under 1 kW/m^2^ solar irradiation was recorded (Figure 6c). Within 1.5 h, all NaCl particles disappeared from the surface, demonstrating the efficient salt rejection capability (efficiently dissolving surface salt deposits) of H2. The deposited salt crystals were observed to dissolve and reabsorb into the hydrogel surface within approximately 1.5 h, confirming a self-cleaning mechanism. This behavior is governed by a combination of physical transport mechanisms. Capillary action continuously draws fresh water through the hydrogel pores to the surface, assisting in the dissolution of surface salt.^48^ Osmotic gradients between the salt-rich surface and the diluted interior drive ion diffusion inward.^49^ Moreover, thermal convection, driven by localized heating and evaporation, creates Marangoni and buoyancy flows that redistribute the salt.^50^ This self-cleaning behavior highlights the potential of H2 for maintaining prolonged desalination efficiency by effectively mitigating salt accumulation. The evaporation rates of the hydrogel under a solar irradiation of 1 kW/m^2^ exhibited a clear dependence on salinity, with a decreasing trend observed with increasing salt concentration, as shown in Figure 6d. At 5 wt % salinity, the evaporation rate was 2.9 kg/m^2^·h, which decreased to 2.4 kg/m^2^·h at 10 wt % and 2.1 kg/m^2^·h at 20 wt %. Figure 6e shows the mass loss that occurred during the solar evaporation process for varying salt concentrations (5, 10, and 20 wt %) when exposed to 1 kW/m^2^ of solar radiation. A consistent decline in the mass loss could be observed as the salinity increased, with recorded values of 2.9 kg/m^2^ for 5 wt %, 2.4 kg/m^2^ for 10 wt %, and 2.1 kg/m^2^ for 20 wt % after 1 h. This reduction was attributed to the decrease in water activity and vapor pressure. The rate of decline was steeper at lower salinity levels, indicating a diminishing impact at higher concentrations due to saturation effects. Even at 20 wt %, the hydrogel maintained an evaporation rate over 2.1 kg/m^2^·h, indicating a moderate level of salt tolerance and applicability for use in brine treatment and desalination. However, the reduced performance at higher salinities underscored the need for further optimization, such as enhancing the porosity of the hydrogel or incorporating salt-repellent coatings, to improve efficiency in highly saline environments.

In the solar-driven evaporation process, salt crystallization prevailed over salt dissolution. Nevertheless, the structural design of the H2 evaporator, including both large-sized pores and a network of interconnecting micropores, offered adequate space to prevent salt accumulation and clogging within the evaporator channels. The presence of these large pores limited salt deposition on the evaporator’s surface, enabling the downward diffusion of salt ions along the concentration gradient. Downward diffusion occurs as salt ions move from areas of higher concentration (on the hydrogel surface, where salt accumulates during evaporation) to areas of lower concentration (in the bulk brine solution beneath the hydrogel). This diffusion occurs in a homogeneous water solution, assuming that the underlying brine remains well-mixed. This diffusion mechanism effectively minimized the likelihood of significant blockages in the water transport pathways (Figure 6f). Furthermore, the extensive pore network contributed to improved water retention capacity, thereby enhancing the evaporator’s performance and ensuring efficient operation even under high-salinity conditions.

The H2 evaporator was subjected to 20 evaporation cycles with 5 wt % brine to assess the its long-term stability under practical conditions. The system was exposed to 1 kW/m^2^ of solar radiation intensity for 1 h throughout each cycle. As depicted in Figure 7a, the evaporation rate across all 20 cycles remained consistently at approximately 2.9 kg/m^2^·h. The mass loss in the first evaporation cycle and that in the last cycle were almost the same (Figure 7b). Specifically, the mass change after the first cycle reached 3.0 kg/m^2^, while the mass change after the 20th cycle exhibited a slightly reduced value of ∼2.9 kg/m^2^. This marginal decrease in mass loss, amounting to only ∼ 3.3%, suggests that the H2 evaporator exhibits exceptional durability and operational stability over repeated use in a saline environment. This slight reduction in mass loss could be attributed to factors such as the gradual accumulation of nonevaporative residues or minor structural changes in the hydrogel over time. However, the negligible difference between the values of the first and final cycles indicates that the evaporator’s performance remains robust and consistent and it effectively maintains its water transport and evaporation efficiency under brine conditions.

**Figure 7:**
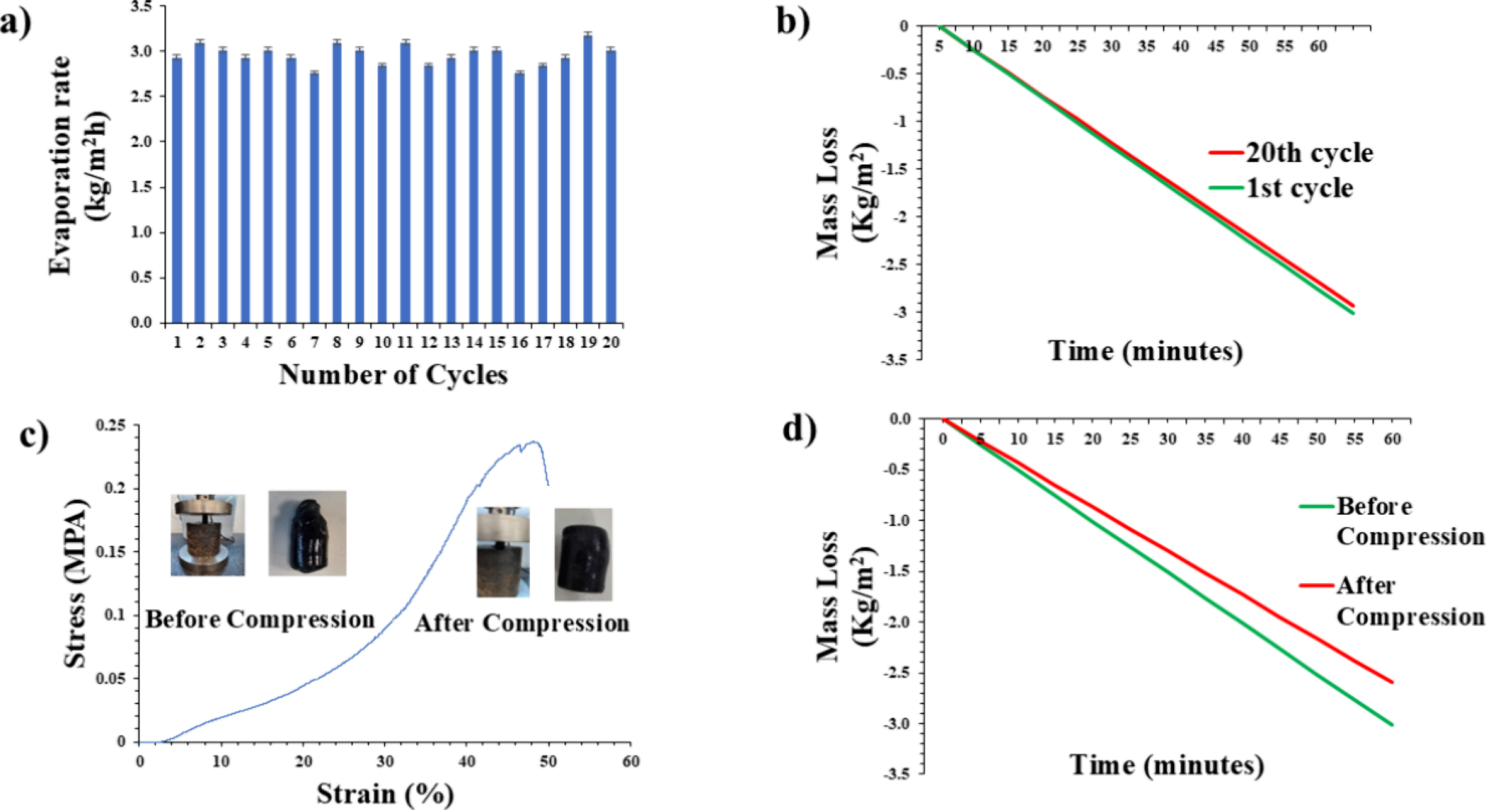
Cyclic durability and mechanical robustness of H2 hydrogel evaporator. (a) Comparison of evaporation rates between first (3.0 kg/m^2^·h) and 20th (2.9 kg/m^2^·h) cycles in 5 wt % brine under 1 sun irradiation, demonstrating only a 3.3% performance degradation. (b) Corresponding mass loss confirming stability across cycles (Δ*m* = 3.0 vs 2.9 kg/m^2^). (c) Compression stress–strain curves showing mechanical resilience (0.23 MPa at 50% strain) due to MWCNT reinforcement. (d) Pre- and postcompression evaporation performance (3.0 → 2.6 kg/m^2^) confirming operational stability under mechanical stress.

Environmental adaptability, durability, and mechanical stability are crucial for the practical application of evaporators. A compression test was conducted to assess the H2 evaporator’s mechanical stability and recovery capabilities. To validate the reinforcement effect of the MWCNTs, uniaxial compression tests were conducted on both H0 and H2. As shown in Figure 7c, the stress–strain curves indicated enhanced mechanical performance for H2 compared to H0 (Figure S6). The compressive modulus of H2 was determined to be 0.0463 MPa, which was significantly greater than that of H0 (0.0278 MPa). The maximum compressive stress before failure for H2 was 0.233 MPa, while H0 failed at 0.192 MPa. Furthermore, the strain of failure increased from 42% in H0 to 49% in H2. These improvements are attributed to the integration of the MWCNTs, which are likely to enhance load transfer and interfacial interactions within the hydrogel network. The results of mass loss of H2 in 5 wt % brine before and after compression under irradiation of 1 kW/m^2^ are depicted in Figure 7d. Prior to compression, the mass loss was recorded to be 3.0 kg/m^2^. After compression, the mass loss decreased slightly to 2.6 kg/m^2^. This minor reduction suggests that while compression may have induced some structural alterations, the overall evaporation performance remained stable. The results indicate that the proposed hydrogel evaporator retains its functionality under mechanical pressure, demonstrating good mechanical resilience and durability for practical solar-driven desalination applications.

Figure 8a illustrates the solar desalination system fabricated using a polyacrylic sheet. The system comprises two distinct compartments, both enclosed by a transparent polyacrylic cover. The first compartment houses seawater and a hydrogel supported by a layer of hydrophilic cotton. The hydrophilic cotton serves as a transport medium, facilitating the movement of seawater into the hydrogel where it is absorbed. Upon absorption, the seawater undergoes evaporation when exposed to sunlight. The resulting water vapor condenses on the inner surface of the polyacrylic cover, forming droplets. These droplets then slide down the inclined cover into the second compartment, which is designated for water collection. This configuration effectively integrates the absorption, evaporation, and condensation processes within a compact and efficient design.

**Figure 8:**
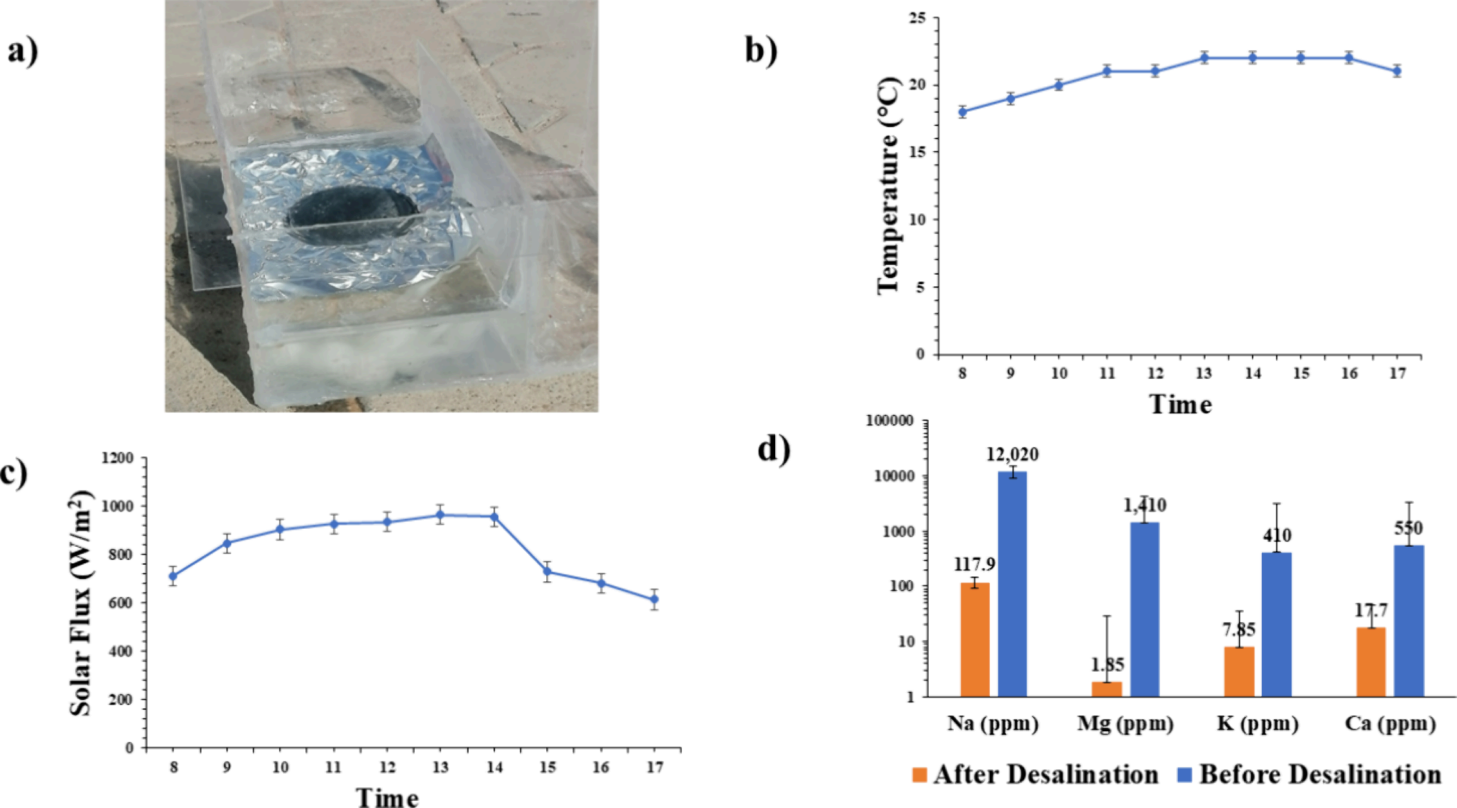
(a) Photograph of the custom-built solar desalination setup used for actual seawater testing. (b) Ambient outdoor temperature variation during the desalination experiment. (c) Change in incident solar flux over time recorded under natural sunlight conditions. (d) Ion concentration in seawater before and after treatment, showing >99% ionic rejection by the H2 hydrogel evaporator.

The economic feasibility of the proposed SA/PVA/Graphite+MWCNT hydrogel evaporator proves to be a key advantage. The hydrogel was prepared using cost-effective materials such as sodium alginate, PVA, graphite, and MWCNTs, avoiding organic solvents or complex synthesis steps. The full solar evaporation system, including insulation, acrylic enclosure, and the transparent top cover, was assembled using locally available materials at a cost effectiveness value of ∼USD 5.5/m^2^, which was calculated using eq S7.^51^

This cost is competitive with or lower than other systems reported in the literature, such as the values reported for a modular bioinspired evaporator (∼USD 15/m^2^) proposed by Guo et al.,^10^ a bilayer hydrogel design (USD 9.18/m^2^) proposed by Du et al.,^52^ and other systems using nanostructured metals or advanced coatings (exceeding USD 50/m^2^).^53,54^

Figure 8b,c depicts the variation in temperature and solar flux during the water desalination process. At the start of the experiment (8:00 AM), the system temperature was recorded to be 18 °C, corresponding to a solar flux of 712 W/m^2^. The temperature gradually increased, reaching its peak value of 22 °C at 1:00 PM when the solar flux was at its maximum of 967 W/m^2^. Toward the end of the experiment, the temperature decreased slightly to 21 °C as the solar flux reduced to 614 W/m^2^. These observations highlight the correlation between solar flux intensity and the thermal behavior of the desalination system throughout the experiment.

Figure 8d presents the ionic concentrations of seawater before and after the desalination process, highlighting the system’s effectiveness in removing ionic impurities. The seawater used in this study was collected from the Abu Dhabi coastline and was analyzed for its initial concentrations of key ions. The concentrations of Na^+^, Mg^2+^, K^+^, and Ca^2+^ were determined to be 12,020 ppm, 1,410, 410, and 550 ppm, respectively. After undergoing the desalination process, there was a substantial reduction in the concentration of all measured ions. Specifically, the Na^+^ concentration decreased to 118 ppm, while the concentration of Mg^2+^ dropped to 1.85 ppm. Similarly, the concentrations of K^+^ and Ca^2+^ were reduced to 7.85 and 17.7 ppm, respectively. A slightly elevated residual concentration of Ca^2+^ ions was observed in the treated water after repeated desalination cycles. This phenomenon is attributed to the partial leaching of Ca^2+^ ions from the hydrogel matrix. Because Ca^2+^ ions were used as ionic cross-linkers to form the SA/PVA hydrogel network, prolonged exposure to brine and heating under solar irradiation may result in the gradual diffusion of free or loosely bound Ca^2+^ ions into the surrounding water. This leaching behavior has also been reported in other ionically cross-linked alginate-based hydrogels^55,56^ and is a known limitation of reversible coordination bonding systems. Secondary filtration using nanofiltration membranes can effectively remove residual Ca^2+.^ ^57,58^ Alternatively, ion exchange resins can be employed to selectively remove Ca^2+^, thereby enhancing the safety of desalinated water.^59,60^ Furthermore, exploring alternative cross-linkers such as genipin^61^ and glutaraldehyde^62,63^ in hydrogel synthesis offers a proactive approach to minimize calcium leaching. Despite this, the concentration of released Ca^2+^ remains within safe limits and does not significantly impact the desalination efficiency. These reductions indicate a removal efficiency exceeding 99% for most ions. The results confirm the high efficiency of the solar-driven desalination system in removing over 99% of ionic impurities, demonstrating its capability to produce water with drastically reduced salinity and mineral content and making it suitable for potable or other nonsaline water applications.

In addition to mechanical durability, the system’s performance under elevated ambient temperatures (e.g., 30, 40, and 50 °C) warrants further investigation. Although this study was conducted at a controlled temperature of ∼23.5 °C, it is expected that higher environmental temperatures may enhance water evaporation due to increased vapor pressure and improved heat transfer at the water–air interface.^5,64^ Previous studies have shown that solar-driven interfacial evaporators exhibit higher evaporation rates at elevated temperatures, particularly in desert-like climates.^11,65^ However, prolonged thermal exposure may also affect the hydrogel’s structural and physicochemical properties, including its swelling behavior, pore stability, and mechanical integrity.^66,67^ Therefore, future studies should evaluate the hydrogel’s thermal tolerance under such conditions to determine its viability for practical solar desalination in high-temperature regions.

## 4 Conclusions

In this study, a porous SA/PVA-based hydrogel evaporator was developed using a simple, environmentally friendly fabrication route combining foaming, freezing, and ionic cross-linking. The integration of graphite and MWCNTs enabled the evaporator to achieve excellent photothermal performance and enhanced structural stability. Unlike many existing evaporators that rely on toxic cross-linkers, which include multistep chemical syntheses, or lack long-term salt management, our system features dual-carbon synergy, salt self-cleaning, and a simple, eco-friendly fabrication route. According to the results, the optimized hydrogel (H2) achieved an evaporation rate of 3.1 kg/m^2^·h with a solar-to-vapor efficiency of 89% under 1 sun irradiation, outperforming many chemically cross-linked and solvent-based evaporators.

More specifically, the evaporator demonstrated intrinsic salt self-cleaning properties: salt deposits from 5 wt % brine resulted in no visible salt residue observed within 1.5 h of solar exposure, and this performance was sustained over 10 h with minimal loss. Additionally, the system showed strong durability, with <3.3% decline in evaporation efficiency after 20 brine cycles. Mechanical compression tests confirmed the resilience of the system under external stress. The hydrogel maintained an evaporation rate >2.1 kg/m^2^·h even at 20 wt % salinity, demonstrating moderate salt tolerance. The evaporator system also demonstrated >99% ionic rejection when tested with real seawater from the UAE coastline, highlighting its practical desalination potential.

While these findings indicate promising short-term stability, further testing over extended durations and with higher salinity levels is needed to completely validate long-term operational durability. The results of this study confirm the novelty of the proposed evaporator in combining hierarchical porosity, dual-carbon photothermal fillers, and a nontoxic, scalable fabrication process. This synergy enables long-term, high-efficiency water purification without complex materials or fabrication steps.

The findings of this study highlight the practicality, low environmental impact, and scalability of the proposed design for real-world decentralized water purification. Future efforts should focus on tuning the hydrogel’s pore architecture to further improve salt rejection under extreme salinity and on scaling the system for field deployment.

## 5 Future Work

While the developed hydrogel system demonstrates promising performance at the laboratory scale, several challenges remain for its large-scale application. Ensuring uniform distribution of photothermal fillers such as MWCNTs and graphite across larger surfaces may require process refinement or advanced dispersion techniques. Additionally, sourcing SA and PVA in industrial quantities with consistent quality could influence batch-to-batch reproducibility. The environmental durability of hydrogel, particularly its resistance to UV degradation, high salinity, and thermal cycling, must be systematically assessed for reliable long-term use in outdoor desalination setups. Addressing these scale-up and stability concerns will be crucial for the commercialization and field deployment of the proposed system.

## Supplementary Material





## Notes

Preparation method for oxidized MWCNTs (Figure S1); additional SEM images at different magnifications (Figures S2–S5); compression test results (Figure S6); statistical comparison data (Table S1); and calculation equations (eqs S1–S7) (PDF)
Video (V1) demonstrating the rapid water absorption of the H2 hydrogel (MP4)

## References

[1] Sharshir S. W., Algazzar A. M., Elmaadawy K. A., Kandeal A. W., Elkadeem M. R., Arunkumar T., Zang J., Yang N. (2020). New Hydrogel Materials
for Improving Solar Water Evaporation, Desalination and Wastewater
Treatment: A Review. Desalination.

[2] Brière, F. G. Drinking-Water Distribution, Sewage, and Rainfall Collection; Presses inter Polytechnique, 2015, p. 548.

[3] Salehi A. A., Ghannadi-Maragheh M., Torab-Mostaedi M., Torkaman R., Asadollahzadeh M. (2021). Hydrogel Materials
as an Emerging Platform for Desalination and the Production of Purified
Water. Sep. Purif. Rev..

[4] Global Water Crisis: The Facts, 2017; CEO Water Mandate. https://ceowatermandate.org/resources/global-water-crisis-facts-2017/ (accessed 2025–10–14).

[5] Chen C., Kuang Y., Hu L. (2019). Challenges and Opportunities for
Solar Evaporation. Joule.

[6] AlMarzooqi F. A., Al Ghaferi A. A., Saadat I., Hilal N. (2014). Application of Capacitive
Deionisation in Water Desalination: A Review. Desalination.

[7] Khawaji A. D., Kutubkhanah I. K., Wie J. M. (2008). Advances in Seawater
Desalination
Technologies. Desalination.

[8] Bamufleh H., Abdelhady F., Baaqeel H. M., El-Halwagi M. M. (2017). Optimization
of Multi-Effect Distillation with Brine Treatment via Membrane Distillation
and Process Heat Integration. Desalination.

[9] Qasim M., Badrelzaman M., Darwish N. N., Darwish N. A., Hilal N. (2019). Reverse Osmosis
Desalination: A State-of-the-Art Review. Desalination.

[10] Guo Y., Lu H., Zhao F., Zhou X., Shi W., Yu G. (2020). Biomass-Derived
Hybrid Hydrogel Evaporators for Cost-Effective Solar Water Purification. Adv. Mater..

[11] Hong S., Shi Y., Li R., Zhang C., Jin Y., Wang P. (2018). Nature-Inspired,
3D Origami Solar Steam Generator toward Near Full Utilization of Solar
Energy. ACS Appl. Mater. Interfaces.

[12] Qiblawey H. M., Banat F. (2008). Solar Thermal Desalination
Technologies. Desalination.

[13] Zhou X., Zhao F., Guo Y., Rosenberger B., Yu G. (2019). Architecting Highly Hydratable Polymer
Networks to Tune the Water
State for Solar Water Purification. Sci. Adv..

[14] Sun Z., Wang M., Mu X., Zhou J., Ke X., Wu Q., Kang M., Wang X., Miao L. (2023). Sustainable β-Cyclodextrin
Modified Polyacrylamide Hydrogel for Highly Efficient Solar-Driven
Water Purification. Mater. Today Energy.

[15] Wang Z., Wu X., Dong J., Yang X., He F., Peng S., Li Y. (2022). Porifera-Inspired
Cost-Effective and Scalable “Porous Hydrogel
Sponge” for Durable and Highly Efficient Solar-Driven Desalination. Chemical Engineering Journal.

[16] Li J., Yan L., Li X., Song W., Li Y. (2022). Porous Polyvinyl Alcohol/Biochar
Hydrogel Induced High Yield Solar Steam Generation and Sustainable
Desalination. J. Environ. Chem. Eng..

[17] Ko Y., Jeong H. Y., Kwon G., Kim D., Lee C., You J. (2020). PH-Responsive
Polyaniline/Polyethylene Glycol Composite Arrays for
Colorimetric Sensor Application. Sens Actuators
B Chem..

[18] Sun M., Yang L., Du X., Gao Y., Zhou X., Sun B., Lyu L. (2024). Biodegradable
Hydrogel with Excellent Portability,
Stability and Anti-Pollution Performance in Solar-Driven Seawater
Desalination. Appl. Therm Eng..

[19] Zhang X., Zhou S., Wang Z., Wei X., Zhang S., Jin J. (2023). Facile Preparation of Hydrogel-Coated
Surfaces with Antifouling and
Salt Resistance for Efficient Solar-Driven Water Evaporation. ACS Appl. Mater. Interfaces.

[20] Merakchi A., Bettayeb S., Drouiche N., Adour L., Lounici H. (2019). Cross-Linking
and Modification of Sodium Alginate Biopolymer for Dye Removal in
Aqueous Solution. Polym. Bull..

[21] Gombotz W. R., Wee S. F. (1998). Protein Release
from Alginate Matrices. Adv. Drug Deliv Rev..

[22] Mo̷rch Ý. A., Donati I., Strand B. L., Skjåk-Bræk G. (2006). Effect of
Ca2+, Ba2+, and Sr2+ on Alginate Microbeads. Biomacromolecules.

[23] Malektaj H., Drozdov A. D., de Claville Christiansen J. (2023). Mechanical Properties
of Alginate Hydrogels Cross-Linked with Multivalent Cations. Polymers.

[24] Wang W., Wang Y., Zheng J., Yu X., Chen W., Li J., Liu Y. N. (2022). A Vasculatural Hydrogel Combined with Prussian Blue
for Solar-Driven Vapor Generation. J. Mater.
Chem. A Mater..

[25] Guo Y., Zhao F., Zhou X., Chen Z., Yu G. (2019). Tailoring
Nanoscale Surface Topography of Hydrogel for Efficient Solar Vapor
Generation. Nano Lett..

[26] Zhu Q., Liu M., Li Y., Feng G., Xu Z., Geng L., Yu X. (2025). Improving
Solar Desalination in High-Salinity Brine through Zwitterionic
Salt Containing Hydrogels. Sep Purif Technol..

[27] Shu L., Zhang X. F., Wang Z., Liu J., Yao J. (2024). Cellulose-Based
Bi-Layer Hydrogel Evaporator with a Low Evaporation Enthalpy for Efficient
Solar Desalination. Carbohydr. Polym..

[28] Deng Z., Miao L., Liu P. F., Zhou J., Wang P., Gu Y., Wang X., Cai H., Sun L., Tanemura S. (2019). Extremely
High Water-Production Created by a Nanoink-Stained PVA Evaporator
with Embossment Structure. Nano Energy.

[29] Yang M., Zhang L., Ye D., Dong Y., Zhan Y., Jiang X. (2024). Facile Preparation
of Sodium Alginate/Poly­(Vinyl Alcohol)/Graphite
Hybrid Porous Hydrogel for Efficient Solar Desalination. Chemical Engineering Journal.

[30] Guo Y., Han Y., Cao Y., Chen Y., Xie J., Ding H., Liang S., Liu X., Sun W., Tang J., Shao S., Xiang J., Shen Y. (2025). Facile Fabrication
of Tough Super Macroporous Hydrogel via Enhanced Phase Separation. Adv. Funct. Mater..

[31] Nakka R., Mungray A. A. (2016). Biodegradable and Biocompatible Temperature Sensitive
Triblock Copolymer Hydrogels as Draw Agents for Forward Osmosis. Sep Purif Technol..

[32] Dey K., Agnelli S., Sartore L. (2023). Designing Viscoelastic Gelatin-PEG
Macroporous Hybrid Hydrogel with Anisotropic Morphology and Mechanical
Properties for Tissue Engineering Application. Micro.

[33] Liu T., Jiao C., Peng X., Chen Y. N., Chen Y., He C., Liu R., Wang H. (2018). Super-Strong and Tough Poly­(Vinyl
Alcohol)/Poly­(Acrylic Acid) Hydrogels Reinforced by Hydrogen Bonding. J. Mater. Chem. B.

[34] Chahal S., Hussain F. S. J., Kumar A., Rasad M. S. B. A., Yusoff M. M. (2016). Fabrication, Characterization and
in Vitro Biocompatibility
of Electrospun Hydroxyethyl Cellulose/Poly (Vinyl) Alcohol Nanofibrous
Composite Biomaterial for Bone Tissue Engineering. Chem. Eng. Sci..

[35] Dey K. K., Kumar P., Yadav R. R., Dhar A., Srivastava A. K. (2014). CuO Nanoellipsoids
for Superior Physicochemical Response of Biodegradable PVA. RSC Adv..

[36] Yue L., Pircheraghi G., Monemian S. A., Manas-Zloczower I. (2014). Epoxy Composites
with Carbon Nanotubes and Graphene Nanoplatelets – Dispersion
and Synergy Effects. Carbon N Y.

[37] Ricciardi R., Auriemma F., De Rosa C., Lauprêtre F. (2004). X-Ray Diffraction
Analysis of Poly­(Vinyl Alcohol) Hydrogels, Obtained by Freezing and
Thawing Techniques. Macromolecules.

[38] El-Nagar H., Abd El-sadek M. S., Ibrahim E. M. M., Elnobi S. (2025). Influence of Multi-Walled
Carbon Nanotubes on Structural and Linear/Nonlinear Optical Properties
of PVA/TiO2 Films for Flexible Optoelectronic Devices. J. Mater. Sci.: Mater. Electron..

[39] Bennettand H., Wiley G. J. O., Benninghoven A., Janssen K. T. F., Tumpner J., Wer H. W. (1993). High Resolution
XPS of Organic Polymers: The Scienta
ESCA300 Database (Beamson, G.; Briggs, D.). J. Chem. Educ..

[40] Ferrari A., Robertson J. (2000). Interpretation
of Raman Spectra of Disordered and Amorphous
Carbon. Phys. Rev. B.

[41] Cullity, B. D. ; Stock, S. R. Elements of X-Ray Diffraction, 3rd ed.; Prentice-Hall, 2001. https://www.scholars.northwestern.edu/en/publications/elements-of-x-ray-diffraction-third-edition (accessed 2025–07–18).

[42] Liang X., Zhong H. J., Ding H., Yu B., Ma X., Liu X., Chong C. M., He J. (2024). Polyvinyl Alcohol (PVA)-Based Hydrogels:
Recent Progress in Fabrication, Properties, and Multifunctional Applications. Polymers.

[43] Liu Z., Wu B., Zhu B., Chen Z., Zhu M., Liu X. (2019). Continuously
Producing Watersteam and Concentrated Brine from Seawater by Hanging
Photothermal Fabrics under Sunlight. Adv. Funct
Mater..

[44] Fan Y., Wang S., Wang F., He J., Tian Z., Zhao H., Zhu Z., Sun H., Liang W., Li A. (2020). The Assembly of a Polymer
and Metal Nanoparticle Coated Glass Capillary
Array for Efficient Solar Desalination. J. Mater.
Chem. A Mater..

[45] Singh S., Shauloff N., Jelinek R. (2019). Solar-Enabled Water Remediation via
Recyclable Carbon Dot/Hydrogel Composites. ACS
Sustain Chem. Eng..

[46] Chen S., Ma D., Gao W., Zhou S., Guo Y., Pan Q., Shuai Y. (2022). High Efficiency Solar Steam Generator
Comprising Sodium Alginate-Polydopamine
Hydrogel for Photothermal Water Sanitation. Sustainable Energy Technologies and Assessments.

[47] Li J., Yao J., Wang X., Wang Y., Huang Y., Ou J., Zheng X., Geng S., Xie C., Wang L. (2025). Super Hydrophilic
Biochar-Based Hydrogel with Tunable Aqueous State for Efficient Solar-Powered
Desalination. Sep Purif Technol..

[48] Li Y., Wang X., Wu R., Qin J., Fu Y., Qin M., Zhang Y., Xu C. (2023). Natural Multiscale
Channeled Salt
Resistant Solar Evaporator for Efficient and Long-Term Water Desalination. Ind. Crops Prod.

[49] Xue Y., Xu H., Long H. (2025). Janus Hydrogels: Advanced Fabrication
Techniques and
Versatile Applications in Solar Evaporation, Biomedicine, and Electronic/Strain
Sensors. ACS Appl. Polym. Mater..

[50] Zhao F., Zhou X., Shi Y., Qian X., Alexander M., Zhao X., Mendez S., Yang R., Qu L., Yu G. (2018). Highly Efficient Solar
Vapour Generation via Hierarchically Nanostructured
Gels. Nat. Nanotechnol..

[51] Eltigani H., Chobaomsup V., Boonyongmaneerat Y. (2024). Cost Effective Photothermal Materials
Selection for Direct Solar-Driven Evaporation. ACS Omega.

[52] Du H., Li Y., Meng J., Wei R., Meng Q., Cao Y., Cui N., Liu H., Yang H. (2025). A Cost-Effective, Salt-Resistant
and Environmentally Stable Solar Evaporator with a Wetting-Gradient
Bilayer Structure for Long-Term Seawater Desalination. Chemical Engineering Journal.

[53] Huang Q., Du C., Huang C. (2022). Nature-Inspired
Pyramid-Shaped 3-Dimensional Structure
for Cost-Effective Heat-Localized Solar Evaporation with High Efficiency
and Salt Localization. Appl. Therm Eng..

[54] Liu Z., Yang Z., Huang X., Xuan C., Xie J., Fu H., Wu Q., Zhang J., Zhou X., Liu Y. (2017). High-Absorption
Recyclable Photothermal Membranes Used in a Bionic System for High-Efficiency
Solar Desalination via Enhanced Localized Heating. J. Mater. Chem. A Mater..

[55] Lee K. Y., Mooney D. J. (2012). Alginate: Properties
and Biomedical Applications. Prog. Polym. Sci..

[56] Kuo C. K., Ma P. X. (2008). Maintaining Dimensions and Mechanical Properties of Ionically Crosslinked
Alginate Hydrogel Scaffolds in Vitro. J. Biomed
Mater. Res. A.

[57] Zhu L., Granda C. B., Holtzapple M. T. (2011). Prevention
of Calcium Sulfate Formation
in Seawater Desalination by Ion Exchange. Desalination
Water Treat.

[58] Molinari R., Avci A. H., Curcio E., Domene D. S., Villa
González C., Gallart J. J. E., Argurio P. (2024). Selective Calcium Removal
at Near-Ambient Temperature in a Multimineral Recovery Process from
Seawater Reverse Osmosis Synthetic Brine and Ex Ante Life Cycle Assessment. Water (Switzerland).

[59] Dahmani K., Kherroub D. E., Boucherdoud A., Bestani B., Dahmani K., Kherroub D. E. (2021). Removal of Ca­(II)
and Mg­(II) Hardness by Ion Exchange
Resins and Soda Ash for Seawater Pretreatment to Reduce Scale Formation
in Evaporators Multi-Stage Flash Desalination. Desalin. Water Treat..

[60] Bornak, B. Desalination by Ion Exchange; Wiley Blackwell, 2014; Vol. 6, pp. 503–520.

[61] Liang H. C., Chang W. H., Liang H. F., Lee M. H., Sung H. W. (2004). Crosslinking
Structures of Gelatin Hydrogels Crosslinked with Genipin or a Water-Soluble
Carbodiimide. J. Appl. Polym. Sci..

[62] Ou A., Bo I. (2017). Chitosan Hydrogels
and Their Glutaraldehyde-Crosslinked Counterparts
as Potential Drug Release and Tissue Engineering Systems - Synthesis,
Characterization, Swelling Kinetics and Mechanism. J. Phys. Chem. Biophys..

[63] Mugnaini G., Gelli R., Mori L., Bonini M. (2023). How to Cross-Link Gelatin:
The Effect of Glutaraldehyde and Glyceraldehyde on the Hydrogel Properties. ACS Appl. Polym. Mater..

[64] Shang W., Deng T. (2016). Solar Steam Generation: Steam by Thermal Concentration. Nat. Energy.

[65] Tao P., Ni G., Song C., Shang W., Wu J., Zhu J., Chen G., Deng T. (2018). Solar-Driven Interfacial Evaporation. Nat.
Energy.

[66] Yang D. (2022). Recent Advances
in Hydrogels. Chem. Mater..

[67] Liu P., Lin W., Wieduwild R., Towers R., Thomas A. K., Günther M., Butdayev S., Wobus M., Bornhäuser M., Zhang Y. (2021). Displaying Lipid Chains in a Peptide-Polysaccharide-Based Self-Assembled
Hydrogel Network. Chem. Mater..

