# Supplementary material for: Self-Cleaning Alginate–PVA Hydrogel Evaporator with Enhanced Solar Desalination Efficiency and Long-Term Salt Resistance
Source: ACS Omega. 2025 Oct 29;10(44):52459–72. doi: 10.1021/acsomega.5c05414 (PMC12612875; doi:10.1021/acsomega.5c05414)
Supplement: Supplementary file 1 [file ao5c05414_si_001.pdf]

Self-Cleaning Alginate–PVA Hydrogel Evaporator with Enhanced Solar Desalination Efficiency and Long-Term Salt Resistance

Muhammad F. Siddique [a], Farag K Omar [a*], Muhammad Waseem [b], Ali H. Al-Marzouqi [b]
[a] Mechanical and Aerospace Engineering Dept, College of Engineering, UAE University, UAE.
[b] Chemical & Petroleum Engineering Dept, College of Engineering, UAE University, UAE.
* Corresponding Author
Email address: fomar@uaeu.ac.ae

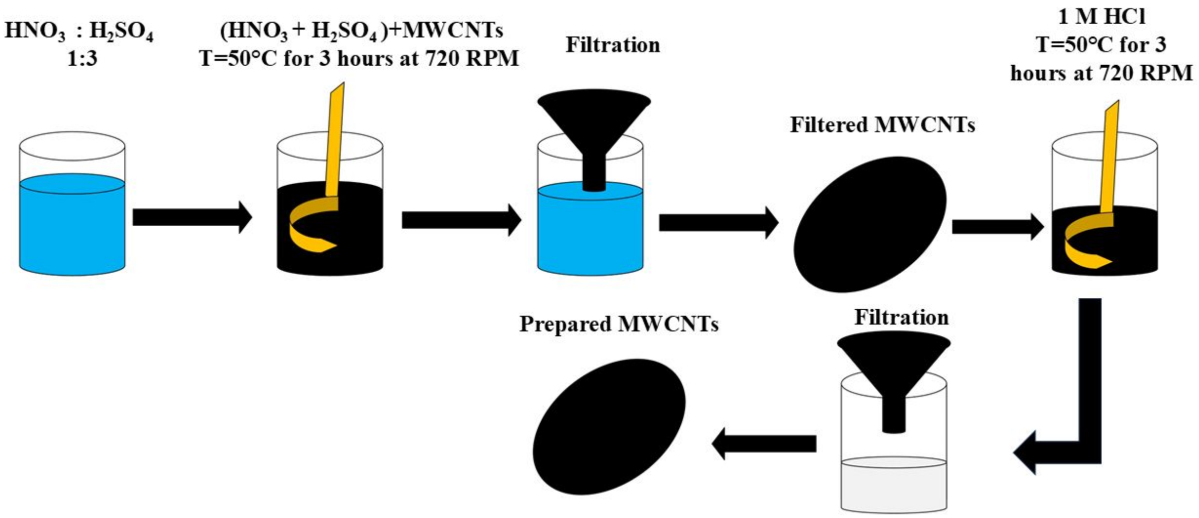

Fig S1. Preparation Method for oxidized MWCNTs

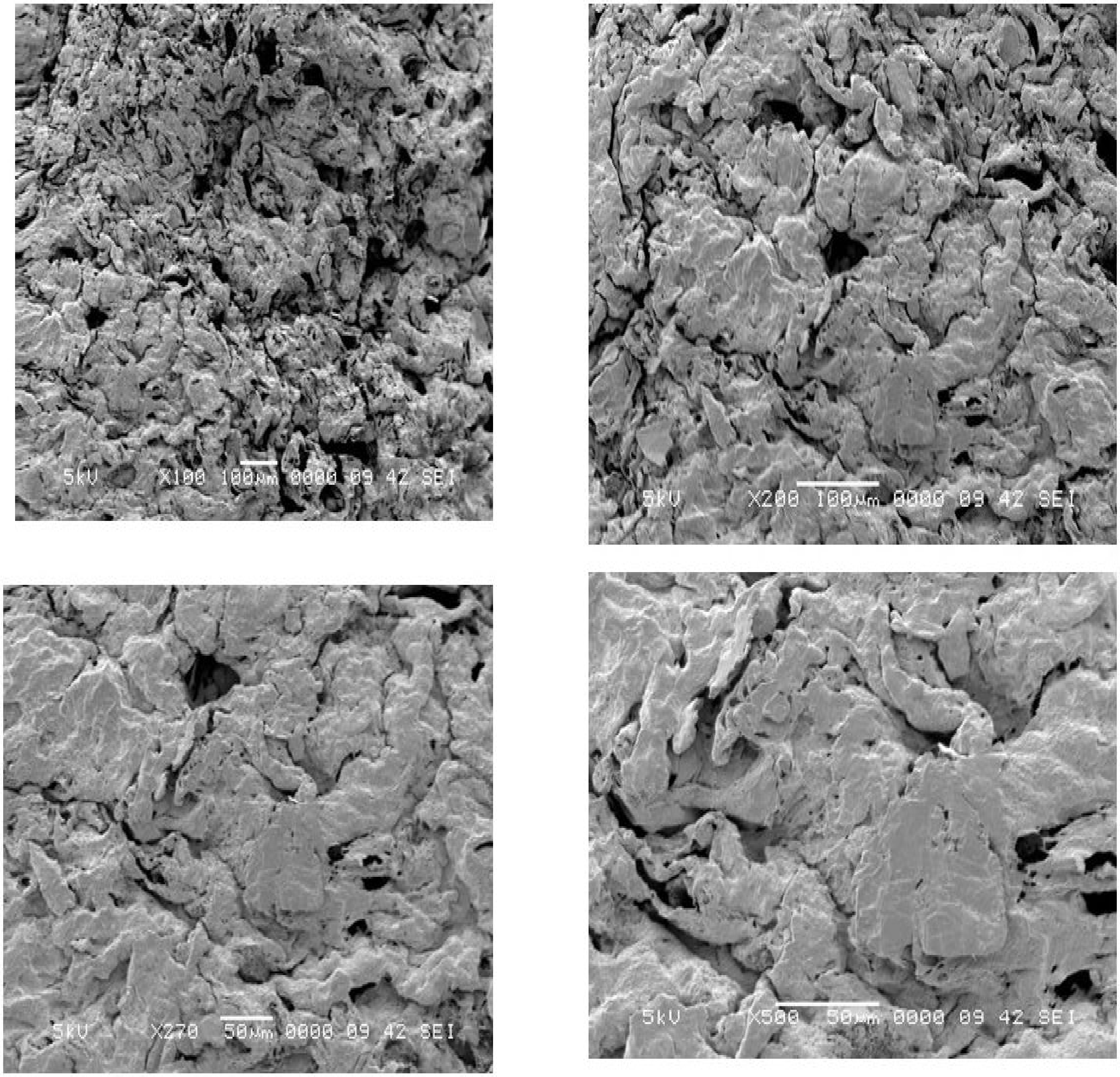

Fig S2. SEM images of H0

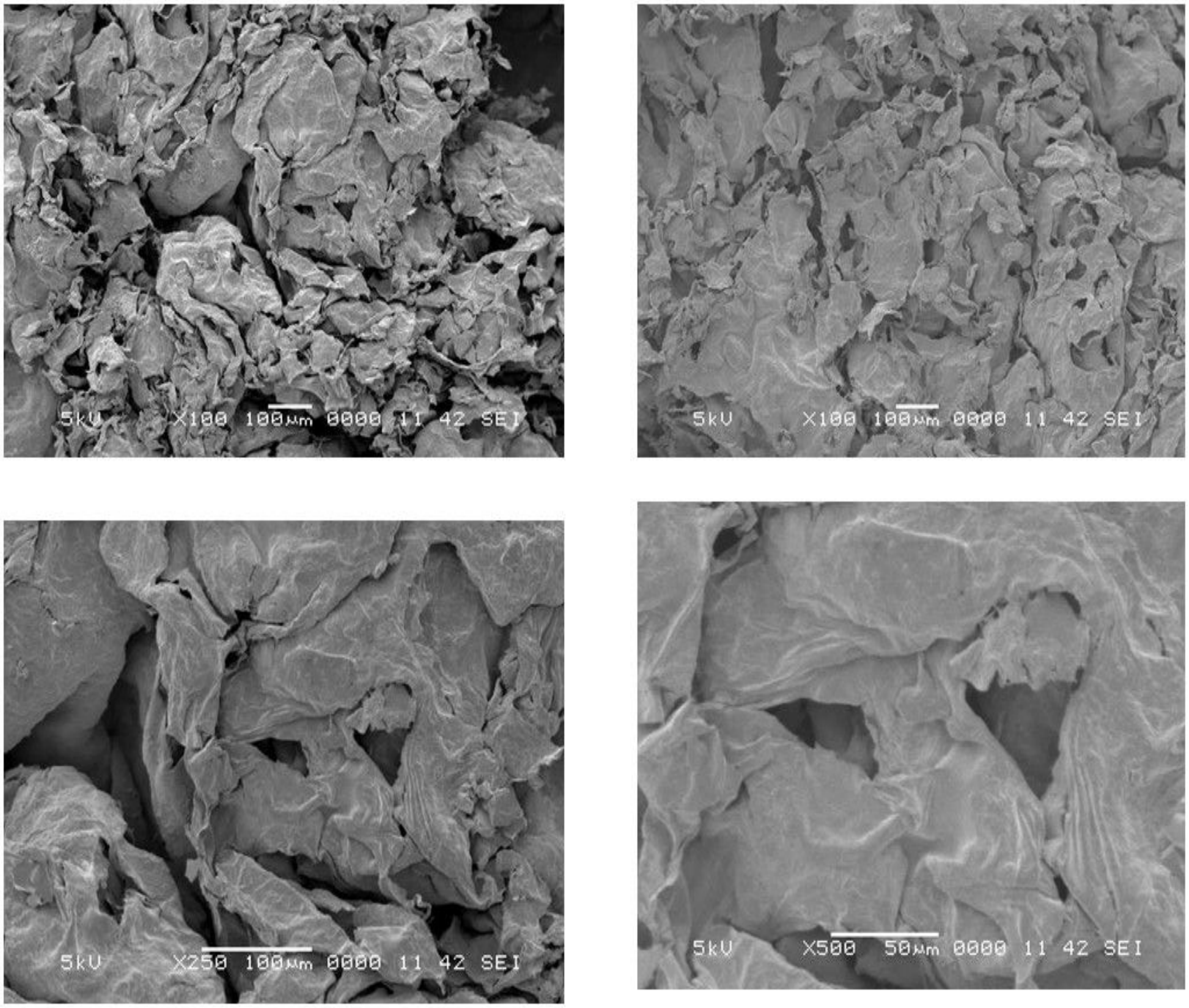

Fig S3. SEM images of H1

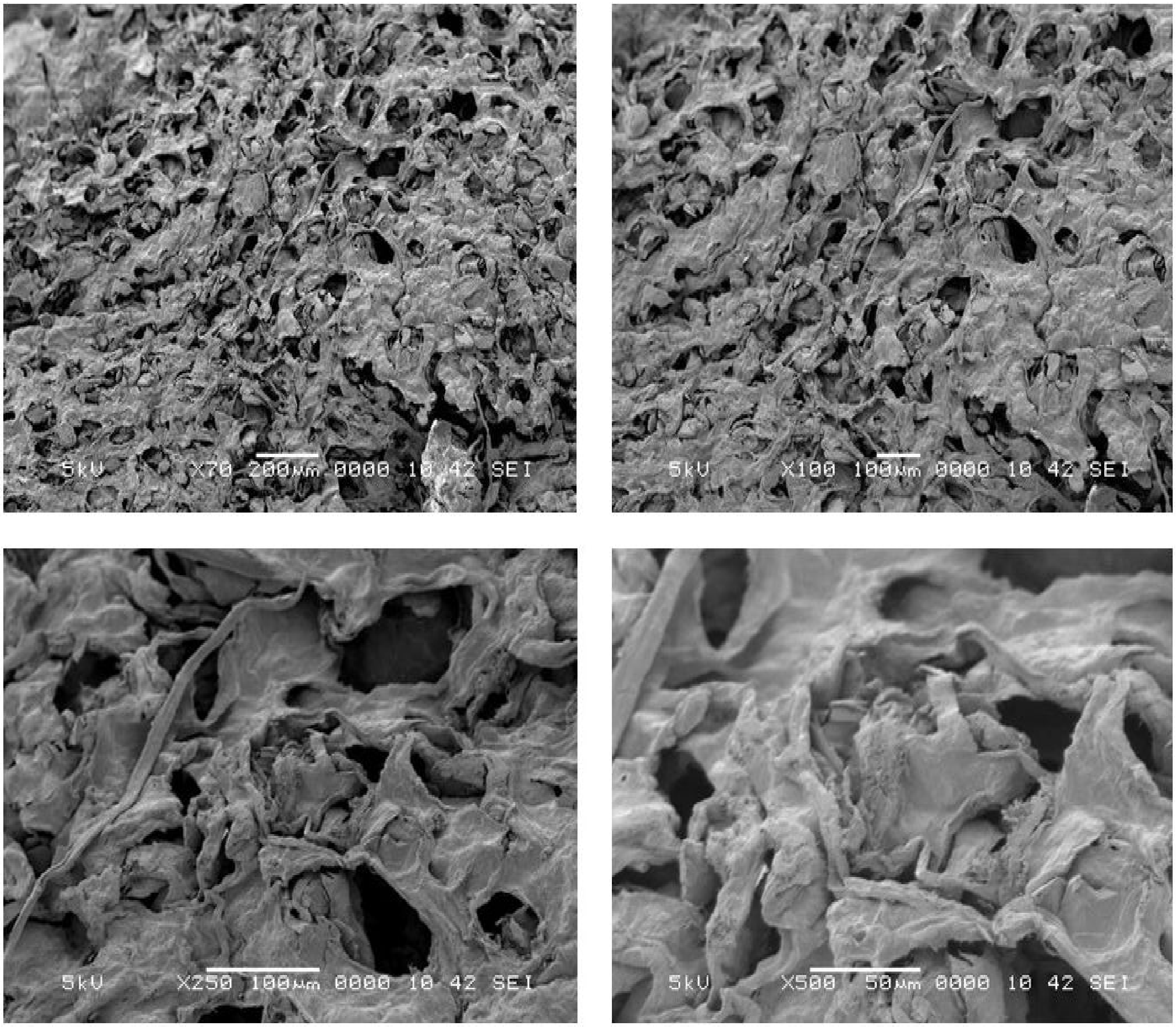

Fig S4. SEM images of H2

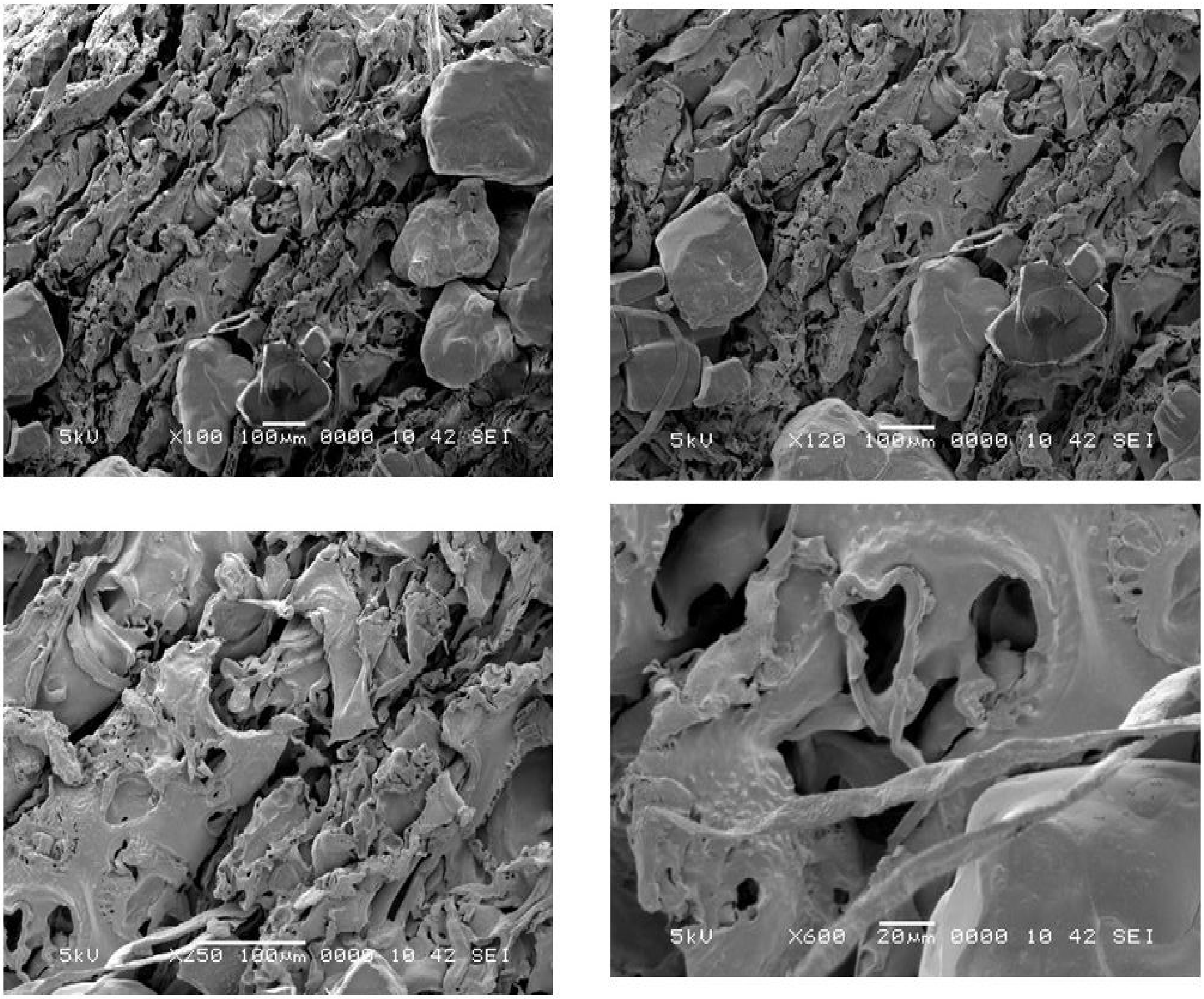

Fig S5. SEM images of H3

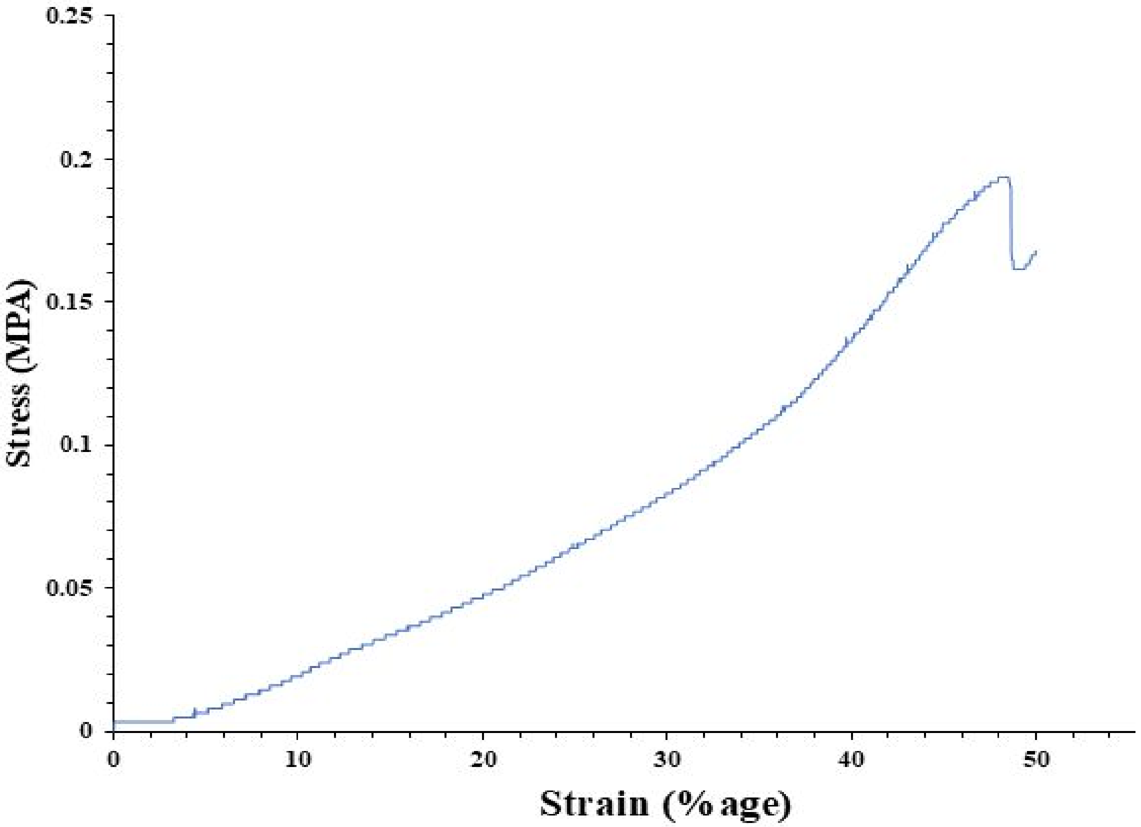

Fig S6. Compression stress-strain curve of H0

Table S1. Statistical Comparison of Evaporation Rates Between Hydrogels

Comparison	t-statistic	Degrees of Freedom	p-value	Significance Level
**H2 vs H1**	27.59	3.43	0.0000	$p < 0.001$
**H2 vs H3**	11.24	3.53	0.0007	$p < 0.001$

Equations

$$\text{Porosity (\%)} = \left(\frac{V_{Total} - V_{Solid}}{V_{Total}}\right) \times 100 \quad \ldots\ldots\ldots\ldots\ldots\ldots \text{(S1)}$$

Where;

V_{Total}= Volume of fully swollen hydrogel

V_{Solid}=Volume of dried hydrogel

$$D = \frac{K\lambda}{\beta cos\theta} \quad \ldots\ldots\ldots\ldots\ldots\ldots \text{(S2)}$$

Where,

D=Average crystallite size

K = Scherrer constant = 0.9

λ= Cu Kα radiation = 0.15406 nm

β = full width at half maximum in radians

θ = Bragg angle

$$Q_s = \frac{(m - m_o)}{m} \quad \ldots\ldots\ldots\ldots\ldots\ldots \text{(S3)}$$

Where,
m_o = Dry weight of the Hydrogel and m is the mass of the fully swollen hydrogel
m = Weight obtained by keeping the hydrogel in water for 12 hours

$$\eta = \frac{(v - v_d)\Delta H_v}{P} \times 100 \quad \ldots\ldots\ldots\ldots\ldots \text{(S4)}$$

$$v = \frac{\Delta m}{St} \quad \ldots\ldots\ldots\ldots\ldots\ldots\ldots\ldots\ldots \text{(S5)}$$

Whereas

Δm = Mass of evaporated water (kg),

t = Time of solar evaporation (h),

S (m^2)= Effective evaporation area (m^2)

P = Irradiation intensity of the Xenon lamp (W/m^2), P=1000 W/m^2

υ_d = Evaporation rate under dark conditions ($kg/m^2 \cdot h$),

ΔHv=water evaporation enthalpy under hydrogel contact.

Eq. (6) can be used to determine ΔHv,

$$m_0.\Delta H_0 = m_{v.}\Delta H_V \ldots\ldots\ldots\ldots\ldots\ldots \text{(S6)}$$

Where,

m_v = mass loss related to the hydrogels,

whereas ΔH_o and m_o stand for the theoretical enthalpy of evaporation (about 2422 J/g) and the mass loss of pure water, respectively.

$$\mathcal{E} = \frac{r}{c} \times 100 \ldots\ldots\ldots\ldots\ldots\ldots \text{(S7)}$$

Where Ɛ (cost effectiveness) in USD/m^2, r refers to the evaporation rate (kg/m^2 h) and c is the cost of raw materials (USD).
